# Cobalt mediates stage-specific toxicity of metal mixtures in cardiovascular–kidney–metabolic syndrome

**DOI:** 10.1093/toxsci/kfaf168

**Published:** 2025-12-12

**Authors:** Wei Zhang, GuangYu Jiang, Ziyan Liu, LianRui Duan, JiaYi Liang, Ziyan Wang, Huiwen Kang, Danyang Huang, Ai Gao

**Affiliations:** Department of Occupational Health and Environmental Health, School of Public Health, Capital Medical University, Beijing 100069, China; Beijing Key Laboratory of Environment and Aging, Capital Medical University, Beijing 100069, China; Department of Occupational Health and Environmental Health, School of Public Health, Capital Medical University, Beijing 100069, China; Department of Occupational Health and Environmental Health, School of Public Health, Capital Medical University, Beijing 100069, China; Department of Occupational Health and Environmental Health, School of Public Health, Capital Medical University, Beijing 100069, China; Department of Occupational Health and Environmental Health, School of Public Health, Capital Medical University, Beijing 100069, China; Department of Occupational Health and Environmental Health, School of Public Health, Capital Medical University, Beijing 100069, China; Department of Occupational Health and Environmental Health, School of Public Health, Capital Medical University, Beijing 100069, China; Department of Occupational Health and Environmental Health, School of Public Health, Capital Medical University, Beijing 100069, China; Department of Occupational Health and Environmental Health, School of Public Health, Capital Medical University, Beijing 100069, China; Beijing Key Laboratory of Environment and Aging, Capital Medical University, Beijing 100069, China

**Keywords:** cardiovascular–kidney–metabolic, metal exposure, cobalt, machine learning, adverse outcome pathway

## Abstract

Cardiovascular–Kidney–Metabolic (CKM) syndrome imposes a rising global health burden, yet the link between environmental metal mixtures and CKM progression remains unclear. To assess the joint effects of metal mixtures on CKM syndrome staging and identify critical toxic drivers through advanced mixture analysis. National Health and Nutrition Examination Survey data (2011 to 2016) from 1,816 participants were analyzed via Weighted Quantile Sum (WQS) regression, generalized linear models (GLMs), ridge regression, Shapley Additive exPlanations (SHAP) analysis, and polynomial regression. An adverse outcome pathway (AOP) framework was utilized to characterize the mechanisms of metal-mediated CKM. The WQS model revealed an association between mixed metal exposure and CKM (β = 0.502, *P* = 0.013). Subsequently, GLMs and ridge regression further identified the associative characteristics of individual metals, with all 3 models pointing to cobalt as the key driver. The SHAP model validated cobalt’s dominant contribution from the perspective of marginal feature importance. Additionally, a polynomial equation analysis showed that cobalt exhibited a linear dose–response relationship with CKM syndrome. Based on these findings, the AOP framework further identified that early CKM stages are linked with cobalt-related metabolic and immune dysregulation. In contrast, late stages involve disruptions in calcium homeostasis, lipid metabolism, and cell apoptosis–survival balance. Our findings highlight the impact of metal exposure on the progression of CKM syndrome; the AOP framework has deciphered stage-specific mechanisms of cobalt, revealing distinct toxicological pathways in early versus late CKM.

## Graphical abstract

**Figure:**
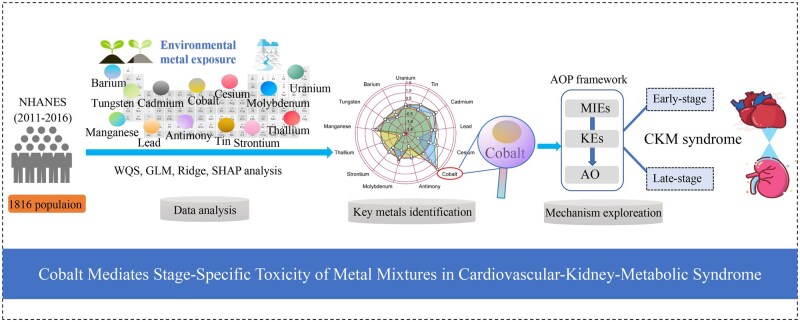

The American Heart Association (AHA) recently established cardiovascular–kidney–metabolic (CKM) syndrome as a clinical entity characterized by interconnected multiorgan dysfunction arising from bidirectional pathological crosstalk between metabolic dysregulation, chronic kidney disease (CKD), and cardiovascular impairment. The tripartite interplay promotes maladaptive systemic pathways that substantially elevate risks of progressive end-organ damage and major adverse cardiovascular events (Larkin 2023). Considering the complex progression of CKM syndrome, the AHA has put forward an age-stratified screening strategy that primarily focuses on detecting early indicators of overweight/obesity, elevated blood pressure, dyslipidemia, and dysglycemia. For individuals with stage 2 CKM syndrome and higher, who are at an elevated risk, the authors recommend a tiered screening protocol that includes assessments for albuminuria, compromised renal function, coronary artery calcium (CAC) deposition, serum cardiac biomarkers, and echocardiographic evaluation; therefore, the CKM syndrome is divided into 5 stages, namely stages 0 to 4 (Claudel and Verma 2023). Of note, a systematic solution including social, environmental, and economic factors could accelerate CKM syndrome development. Conversely, certain interventions may alleviate the CKM syndrome. Environmental exposure to certain hazardous pollutants could lead to poor CKM syndrome.

Metals have the characteristics of accumulation and antidegradation and have negative effects on the human body, including carcinogenic and noncarcinogenic effects (Briffa et al. 2020). Numerous studies have demonstrated that metal exposure acts as a major independent risk factor for cardiovascular disease (CVD) progression (Pan et al. 2024). In particular, exposure to lead and cadmium independently heightens the likelihood of developing cardiovascular disorders, such as acute myocardial infarction and heart failure. One study also showed that exposure to multiple metals (Cd, Cu, Mg, Mo, and Zn) was linked to an elevated risk of hypertension (Zhong et al. 2021). Meanwhile, 1 study based on the National Health and Nutrition Examination Survey (NHANES) database showed that exposure to metal increases the likelihood of CKD in patients with type 2 diabetes mellitus (T2DM) (Shi et al. 2024). Another study also revealed that metals, including lead and mercury, were prominently correlated with metabolic syndrome (MetS) (Xu et al. 2021). However, the relationship between the metals and CKM is of paramount urgency.

The adverse outcome pathway (AOP) functions as a mechanistic framework to establish causal links between metal exposures and biological endpoints, structured around a sequential chain of molecular initiating events, key events (KEs), and adverse outcomes (Ankley et al. 2010). In this study, using the mixture analysis model and the single-exposure model, we examined the impact of metal mixtures on the progression of CKM syndrome. This multi-model integration study pioneers in decoding the metallomics code of CKM syndrome, identifying cobalt as a threshold-free risk driver through machine learning interpretability. Besides, the AOP further explains the potential toxicity pathways related to the early- and late-stage CKM, respectively. Overall, this study identifies critical associations between metals and CKM progression, providing mechanistic insights into potential toxic pathways underlying the metal–CKM interaction.

## Materials and methods

### Study population

The NHANES is a biennial research initiative aimed at evaluating the health and nutritional status of US adults and children. Study participants provided informed consent regarding the survey procedures and their rights as subjects, and the protocol was approved by the NCHS Review Board (McGraw et al. 2023). In this study, all data came from NHANES (2011 to 2016) with available urine metal exposure and cardio–renal MetS data. Considering the metabolism pattern difference in teenagers and pregnant women, our inclusion criteria were as follows: Age 20 or older, male, female, and non-pregnant. Only subjects with complete data on metals and all covariates (education, ethnicity, body mass index [BMI], gender, age, smoking, alcohol consumption, physical activity, hypertension, and diabetes) were included in this study. Finally, the sample was 1,816 participants. This cross-sectional study utilized data from NHANES, with informed consent obtained from all participants. We extend our sincere gratitude to the NHANES team for their contributions.

### Metal detection

The previous study has depicted the detection of urine metals in detail (McGraw et al. 2023), Inductively coupled plasma-Mass Spectrometry (ICP-MS) was utilized to detect urine metals, including barium, cadmium, cobalt, cesium, molybdenum, manganese, lead, antimony, tin, strontium, thallium, tungsten, and uranium. For results below the lower limit of detection, an imputed value (LLOD/√2) was used according to the description of the NHANES.

### Cardiovascular-Kidney-Metabolic Syndrome definition

The classification framework for Cardiovascular–Kidney–Metabolic (CKM) disease progression (stratified into phases 0 through 4) was established through the adaptation of existing diagnostic protocols (Aggarwal et al. 2024), with methodological adaptations for NHANES dataset compatibility. The detailed procedure is shown in the online supplementary material.

### Concomitant variable

The adjusted covariates included sociodemographic data (age, gender, ethnicity, education level, annual household income, marital status, BMI, physical activity, smoking status, alcohol consumption, diabetes, and hypertension). The detailed characteristic of the concomitant variable is shown in the online supplementary material.

### Data analysis

Urinary metal concentrations were creatinine-adjusted, and we performed a log10-transformed analysis to address skewed distributions. Spearman was used to analyze the correlation of the metal substances. In the descriptive statistical analysis, Chi-square analyses were conducted to determine whether significant differences existed in the distribution of categorical variables between the 2 groups. When the expected frequency of the contingency table is less than 5, the Fisher’s exact test is used as an alternative method. In addition, the Benjamini–Hochberg correction is performed on all *P*-values to deal with potential multiple comparison problems. The interquartile range (IQR) was used to describe the distribution of metals in the early and late stages. All of the analysis was conducted by R (version of R 4.5.0). All analyses took into consideration the complex survey design of NHANES. The sample weight was set as one-third of WTS2YR, where WTS2YR represents the 2-year sample weight during each survey period. The main analytical methods include generalized linear models (GLMs), Weighted Quantile Sum (WQS), Ridge Regression, and Polynomial Regression. K-means Clustering and *t*-distributed Stochastic Neighbor Embedding Visualization. Detailed analysis details have been listed in the online supplementary materials.

### AOP framework construction

As depicted in the previous study (Fan et al. 2024), the workflow of the AOP study was performed and shown in Fig. S1. The CTD database was queried using the keyword “cobalt” to retrieve associated genes and phenotypes. GeneCards (https://www.genecards.org) and DisGeNET (https://www.disgenet.org) were utilized to collect early- and late-stage CKM-related genes. For the early-stage screening, keywords included “type 2 diabetes mellitus (T2DM),” “hypertriglyceridemia,” “metabolic syndrome,” “hypertension,” and “obesity.” The late-stage screening utilized “chronic kidney diseases” and “heart failure” as primary search terms. Gene intersection analyses were subsequently conducted to identify key initiating molecules. In the bioinformatics analysis phase, Gene Ontology (GO) and Kyoto Encyclopedia of Genes and Genomes (KEGG) enrichment analyses were utilized to characterize gene–phenotype interactions, and then intersection with cobalt-related phenotypes in the CTD databases.

## Results

### Basic demographic characteristics of study participants

**Fig. 1.**
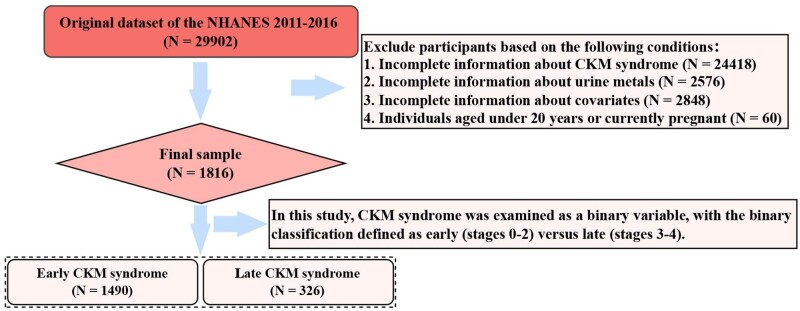
Flowchart depicting the participants in this study.

Figure 1 shows the flowchart of this study, and the final analysis included a total of 1,816 participants. CKM syndrome was divided into early (*n* = 1,490) and late stages (*n* = 326). There were 52.1% males and 47.9% females, respectively. The average age of the participants was divided into 3 stages, including young (32.8%), middle-aged (40.4%), and elderly (26.8%). Besides, Table 1 presents a summary of the baseline characteristics for the study participants; the age, gender, education, race, high blood pressure, and marital status have significant differences in different groups (*P* < 0.05).

**Table 1.** Basic characteristics according to CKM syndrome in the National Health and Nutrition Examination Survey, 2011 to 2016.

Variables	Total (*n* = 1,816)	Early (*n* = 1,490)	Late (*n* = 326)	*P* value
Age (groups)				<0.05
Young	596 (32.8%)	331 (18.2%)	265 (14.6%)	
Middle	734 (40.4%)	679 (37.4%)	55 (3.0%)	
Old	486 (26.8%)	480 (26.4%)	6 (0.3%)	
Sex				<0.05
Male	946 (52.1%)	745 (41.0%)	201 (11.1%)	
Female	870 (47.9%)	745 (41.0%)	125 (6.9%)	
Education level				<0.05
Post_high school	385 (21.2%)	283 (15.6%)	102 (5.6%)	
High school	400 (22.0%)	314 (17.3%)	86 (4.7%)	
After_high school	1031 (56.8%)	893 (49.2%)	138 (7.6%)	
Race/ethnicity				<0.05
Mexican	251 (13.8%)	213 (11.7%)	38 (2.1%)	
Other Hispanic	214 (11.8%)	180 (9.9%)	34 (1.9%)	
White	754 (41.5%)	581 (32.0%)	173 (9.5%)	
Black	348 (19.2%)	287 (15.8%)	61 (3.4%)	
Asian	207 (11.4%)	190 (10.5%)	17 (0.9%)	
Other race	42 (2.3%)	39 (2.1%)	3 (0.2%)	
Poverty				0.596
Poor	1399 (77.0%)	1152 (63.4%)	247 (13.6%)	
Non-poor	417 (23.0%)	338 (18.6%)	79 (4.4%)	
BMI (groups)				0.144
Low_weight	36 (2.0%)	33 (1.8%)	3 (0.2%)	
Normal	507 (27.9%)	428 (23.6%)	79 (4.4%)	
Over_weight	596 (32.8%)	481 (26.5%)	115 (6.3%)	
Obese	677 (37.3%)	548 (30.2%)	129 (7.1%)	
Alcohol consumption				0.309
Yes	1336 (73.6%)	1104 (60.8%)	232 (12.8%)	
No	480 (26.4%)	386 (21.3%)	94 (5.2%)	
Tobacco use				0.496
Yes	453 (24.9%)	377 (20.8%)	76 (4.2%)	
No	1363 (75.1%)	1113 (61.3%)	250 (13.8%)	
Physical activity				0.079
Yes	682 (37.6%)	574 (31.6%)	108 (5.9%)	
No	1134 (62.4%)	916 (50.4%)	218 (12.0%)	
High blood pressure				
Yes	667 (36.7%)	425 (23.4%)	242 (13.3%)	< 0.05
No	1149 (63.3%)	1065 (58.6%)	84 (4.6%)	
Diabetes status				
Yes	1542 (84.9%)	1333 (73.4%)	209 (11.5%)	< 0.05
No	274 (15.1%)	157 (8.6%)	117 (6.4%)	
Marital status				< 0.05
Married	914 (50.3%)	740 (40.7%)	174 (9.6%)	
Widowed	122 (6.7%)	60 (3.3%)	62 (3.4%)	
Divorced	203 (11.2%)	153 (8.4%)	50 (2.8%)	
Separated	56 (3.1%)	50 (2.8%)	6 (0.3%)	
Never married	367 (20.2%)	346 (19.1%)	21 (1.2%)	
Living with partner	154 (8.5%)	141 (7.8%)	13 (0.7%)	

### Distribution and correlation of metals

After log10 conversion, the distribution of 13 heavy metals in the urine was evaluated using the IQR according to the CKM stages, with barium and thallium showing the higher level in early CKM, whereas cadmium, cobalt, lead, tin, and uranium demonstrated the high level in late CKM (*P* < 0.05) (Table 2). Next, by calculating the Spearman correlation coefficient of adjusted residuals, we assessed the correlation between 13 metals. The results revealed that there is a moderate relationship (0.4 < cor < 0.7) between strontium and barium, thallium and cesium, and strontium and cobalt (Fig. S2).

**Table 2.** Descriptive of the quartile distribution of metals in urine according to CKM stages.

Metal	Early			Late			*P* value
	Q1	Median	Q3	Q1	Median	Q3	
Barium	0.002637203	0.004547036	0.007956933	0.00185924	0.003986573	0.008423696	<0.05
Cadmium	0.000518699	0.000941781	0.001795171	0.000903109	0.001653369	0.002914648	<0.05
Cesium	0.012294339	0.016919107	0.024232538	0.013794162	0.017713226	0.024077905	
Cobalt	0.000981212	0.001415357	0.002054927	0.001051361	0.001504031	0.002374934	<0.05
Manganese	0.000309739	0.000494259	0.000822685	0.000322753	0.000475241	0.000825925	
Molybdenum	0.096185344	0.133299752	0.175784802	0.098489713	0.135636879	0.173952501	
Lead	0.000890132	0.001396742	0.002307144	0.001209763	0.001975961	0.002879268	<0.05
Antimony	0.000135589	0.000196377	0.000302013	0.000144943	0.000208112	0.000301451	
Strontium	0.200709761	0.280556538	0.368960414	0.190654407	0.264156175	0.372231794	
Thallium	0.00049716	0.000720493	0.00102432	0.000391454	0.000565035	0.000838185	<0.05
Tin	0.001072676	0.001925921	0.003966061	0.001812029	0.003409323	0.006867849	<0.05
Tungsten	0.000140855	0.000232599	0.000389165	0.00015772	0.000271022	0.000415421	
Uranium	1.34312E-05	2.23625E-05	3.91063E-05	1.52E-05	2.55184E-05	4.89143E-05	<0.05

### The relationship between metal mixture exposure and the CKM syndrome analyzed by the WQS regression model

**Fig. 2.**
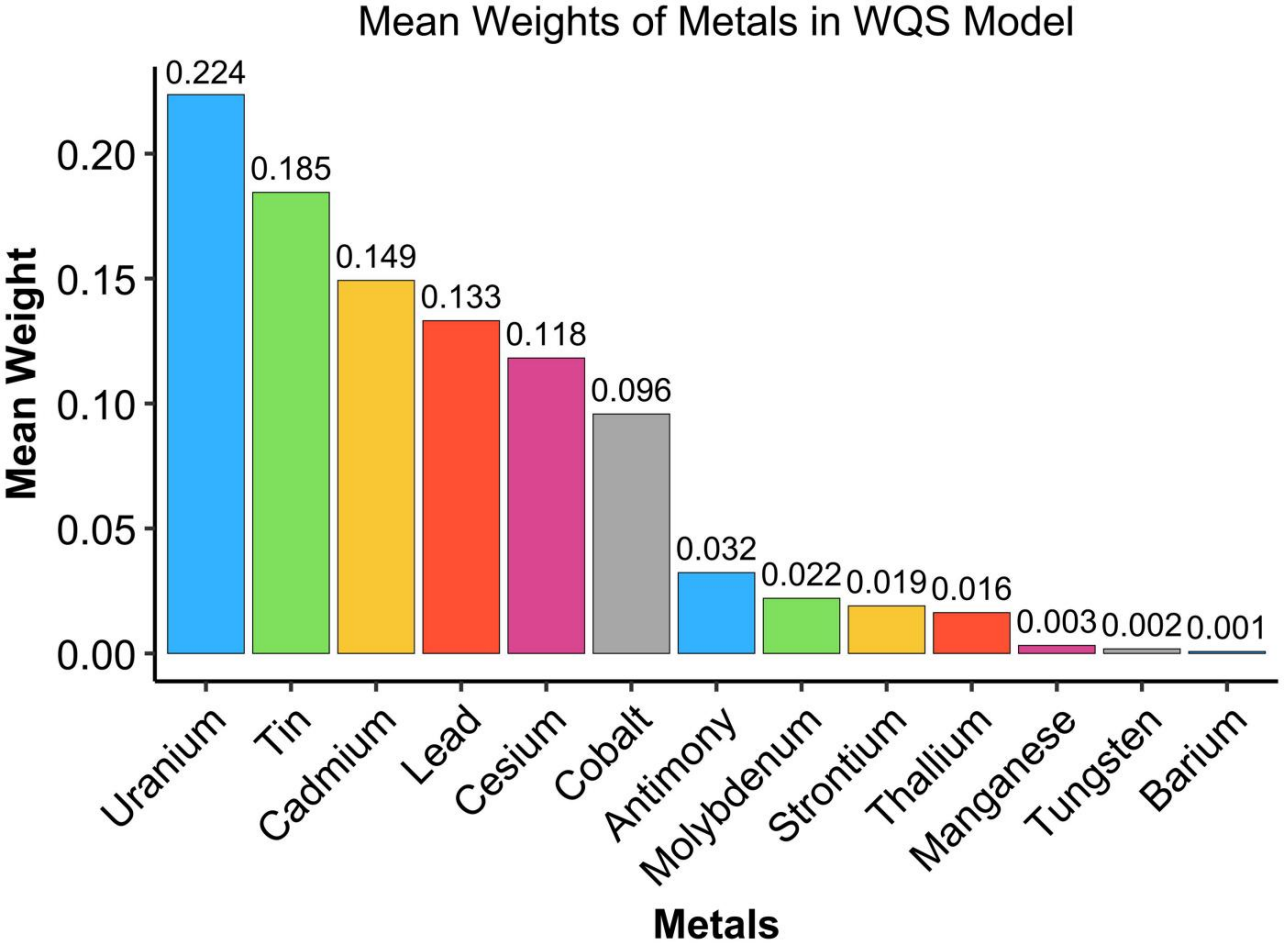
Associations between metal mixture levels and CKM syndrome stages were analyzed via WQS regression, in which individual metal weights impact the aggregate effect.

Using WQS regression, we further explored the association between mixed metal exposure and CKM syndrome. The analysis revealed that a one-quantile increase in the WQS index was linked to a 0.502-unit higher risk of CKM syndrome (β = 0.502, *P* = 0.013). The bar chart in Fig. 2 shows the weight analysis proportion of each metal in the WQS regression analysis, among which uranium, tin, cadmium, lead, cesium, and cobalt are at the forefront after adjusting all of the covariates.

### Associations between single metal exposure and CKM syndrome

**Fig. 3.**
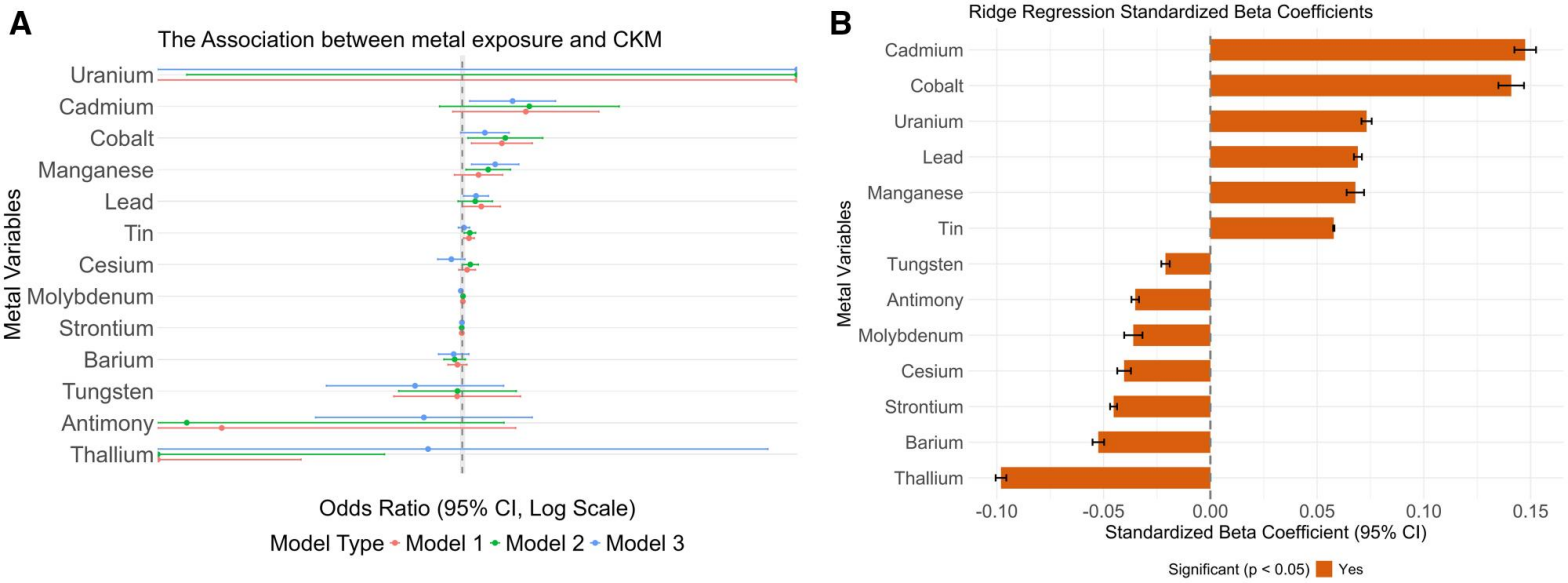
The association analysis between urine metals and CKM syndrome stages using generalized regression models and ridge regression model. A) Generalized regression models, including model 1 (without any adjustment), model 2 (adjusted for gender, education, and ethnicity), and model 3 (adjusted for age, BMI, poverty, alcohol, tobacco, diabetes, physical activity, high blood pressure, and marital status). B) Ridge regression model construction to evaluate the association between metal exposure and CKM syndrome.

On this basis, we constructed a GLM and ridge model to initially analyze whether metal-alone exposure affects the CKM syndrome progress. In the GLM (Fig. 3A), the relationship between metals and CKM syndrome was first evaluated without any covariates included in model 1. The result shows that cobalt (β = 85, *P* < 0.05) and tin (β = 14, *P* < 0.05) positively related to the CKM syndrome stages, whereas thallium (β=−1,028, *P* < 0.05) has a negative influence on the CKM syndrome stages. The relationship continued after adjusting the gender, age, and education of participants in model 2. Cobalt (β = 92, *P* < 0.05), manganese (β = 56, *P* < 0.05), and tin (β = 16, *P* < 0.05) have a positive influence on the CKM syndrome stages, whereas thallium (β=−831, *P* < 0.05) has a negative influence on the CKM syndrome stages. Besides, we evaluate the relationship between metals and CKM syndrome by including all covariates, including gender, age, poverty, education, marital status, BMI, diabetes, high blood pressure, smoking, alcohol consumption, and physical activity, in model 3; cadmium (β = 108, *P* < 0.05), lead (β = 29, *P* < 0.05), and manganese (β = 71, *P* < 0.05). Furthermore, we performed a stratified analysis by smoking status. The results showed in Table S1 that in the non-smoking population, cadmium and manganese exposure were significantly positively associated with the CKM (*P* < 0.05). No similar association was observed in the smoking population, suggesting that smoking status may modify the health effects of these 2 metals.

The ridge regression model further revealed that cobalt (95% CI: 0.142 to 0.153) and cadmium (95% CI: 0.135 to 0.147), whereas thallium (95% CI: −0.101 to −0.011) had a negative on the CKM (Fig. 3B). Overall, the above results indicate the independent role metals in the progression of CKM syndrome.

### Identification of exposure patterns and feature importance via k-means clustering and SHAP analysis

**Fig. 4.**
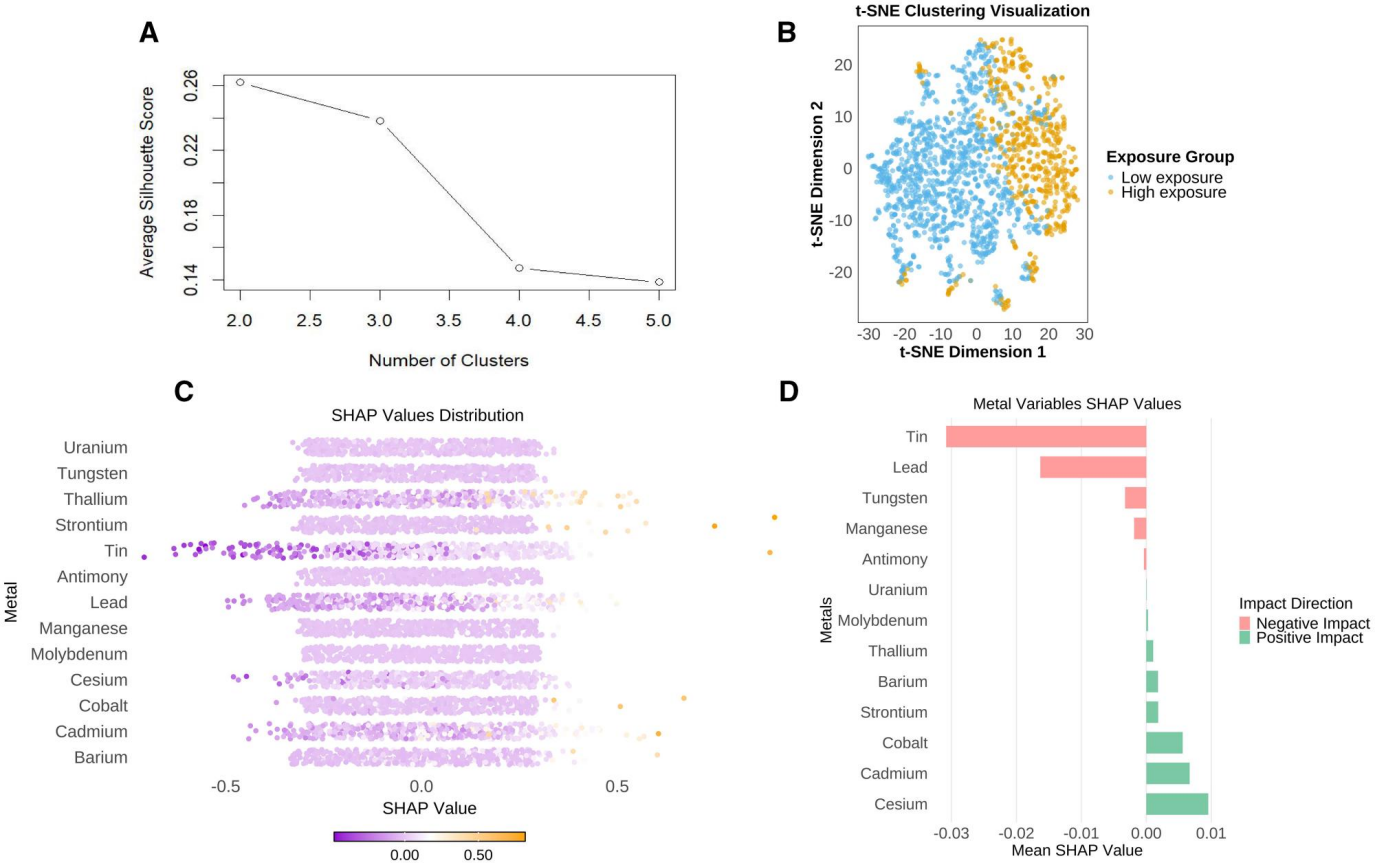
K-means clustering results were visualized by *t*-SNE and SHAP analysis. A) The silhouette score was used to measure intra-cluster compactness and inter-cluster separation. B) The results of k-means clustering of a dataset using the *t*-distributed Stochastic Neighbor Embedding (*t*-SNE) algorithm. C) SHAP honeycomb diagram of the metals across all of the samples. D) The bar chart presents the mean SHAP values of metals for the predictive model.

Next, an integrated machine learning framework combining unsupervised pattern recognition (k-means clustering) with explainable artificial intelligence (Shapley Additive exPlanations [SHAP] analysis) was constructed to observe the interplay between environmental exposure patterns and CKM syndrome. The results in Fig. 4A show that the average score peaked at approximately 0.25 when the number of clusters (k) was 2. Based on this result, samples were divided into 2 exposure groups (Low exposure and High exposure), and *t*-SNE was used to verify the clustering result (Fig. 4B). Subsequently, stratified SHAP analysis was implemented to explore differential feature importance patterns across the 2 groups and all samples. Figure 4C presents a beeswarm plot illustrating the distribution of SHAP values for each metal feature. This plot indicates both the direction (positive or negative) and magnitude of each metal feature’s impact on the model’s predictions regarding CKM syndrome. Figure 4D shows the mean SHAP of the metals. It can be seen that cesium, cadmium, and cobalt have a positive influence on the CKM, whereas tin, lead, and tungsten have a negative association with CKM syndrome.

In addition, we also evaluated the effects of covariates on CKM syndrome. As shown in Fig. S3, age (youth and middle-aged) and the feature of no high blood pressure account for the majority in terms of the average impact.

### Nonlinear associations between metal exposure and CKM syndrome assessed by polynomial regression model

**Fig. 5.**
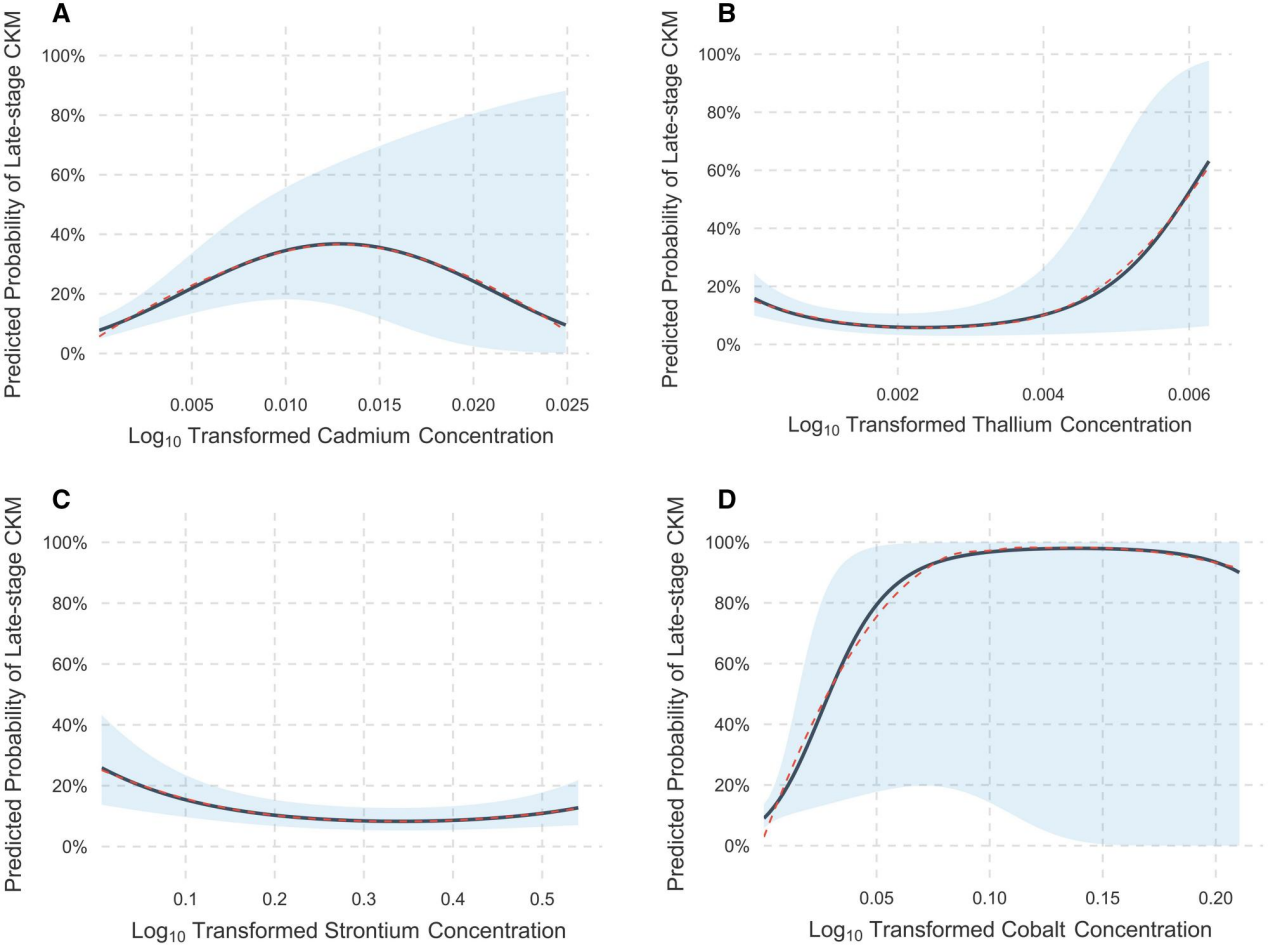
Polynomial regression showed a non-linear relationship between cadmium (A), strontium (B), thallium (C), and CKM stage, whereas cobalt (D) exhibited a significant linear association with the CKM stage.

Furthermore, we evaluated the correlation between the metals and CKM syndrome using polynomial regression. Results showed that the quadratic terms of cadmium (Fig. 5A), strontium (Fig. 5B), and thallium (Fig. 5C) were statistically significant (*P* < 0.05), indicating nonlinear dose–response relationships. Of note, the inflection points were 0.013 μg/g for cadmium, 0.341 μg/g for strontium, and 0.002 μg/g for thallium. Although the quadratic term for cobalt was non-significant (*P* = 0.269), a significant linear association was observed (*P* < 0.05) (Fig. 5D). No significant dose–response relationships were observed for the remaining 9 metals.

### The workflow of AOP construction in cobalt-driven CKM

Integrative analysis of multi-model weights revealed that cobalt consistently exhibited significantly high weights across WQS, Ridge, and GLM models (Fig. S4), with linear relationships supporting its prioritization as a key substance for subsequent mechanistic investigations. Herein, we explored the AOP of cobalt to preliminarily decipher the underlying mechanisms. For the early stage, 1 key molecule (insulin, INS) was identified as the molecular initial event, and the subsequent GO and KEGG analyses identified 315 phenotypes and 31 pathways. In the late stage, 24 key molecules were initially obtained; following literature screening and screening for cardiac/renal expression via the HPA database (Table S2), nitric oxide synthase 3, angiotensinogen, angiotensin I converting enzyme, C–C motif chemokine ligand 2, intercellular adhesion molecule 1, natriuretic peptide B, selectin E, PPARG coactivator 1 alpha, and G protein-coupled receptor kinase 5 were selected as the initial molecular events. Afterwards, GO and KEGG analyses were enriched and revealed 365 phenotypes and 18 pathways. Following phenotype intersection with cobalt-associated entries in the CTD database, literature-based curation was performed to determine the KE chain, integrating all preceding findings. Finally, 247 GO functions (Table S3) and 28 KEGG pathways (Table S4) were selected for early CKM, whereas 208 GO functions (Table S5) and 16 KEGG pathways (Table S6) were selected for the late CKM.

### Constructing the cobalt-related AOP framework

**Fig. 6.**
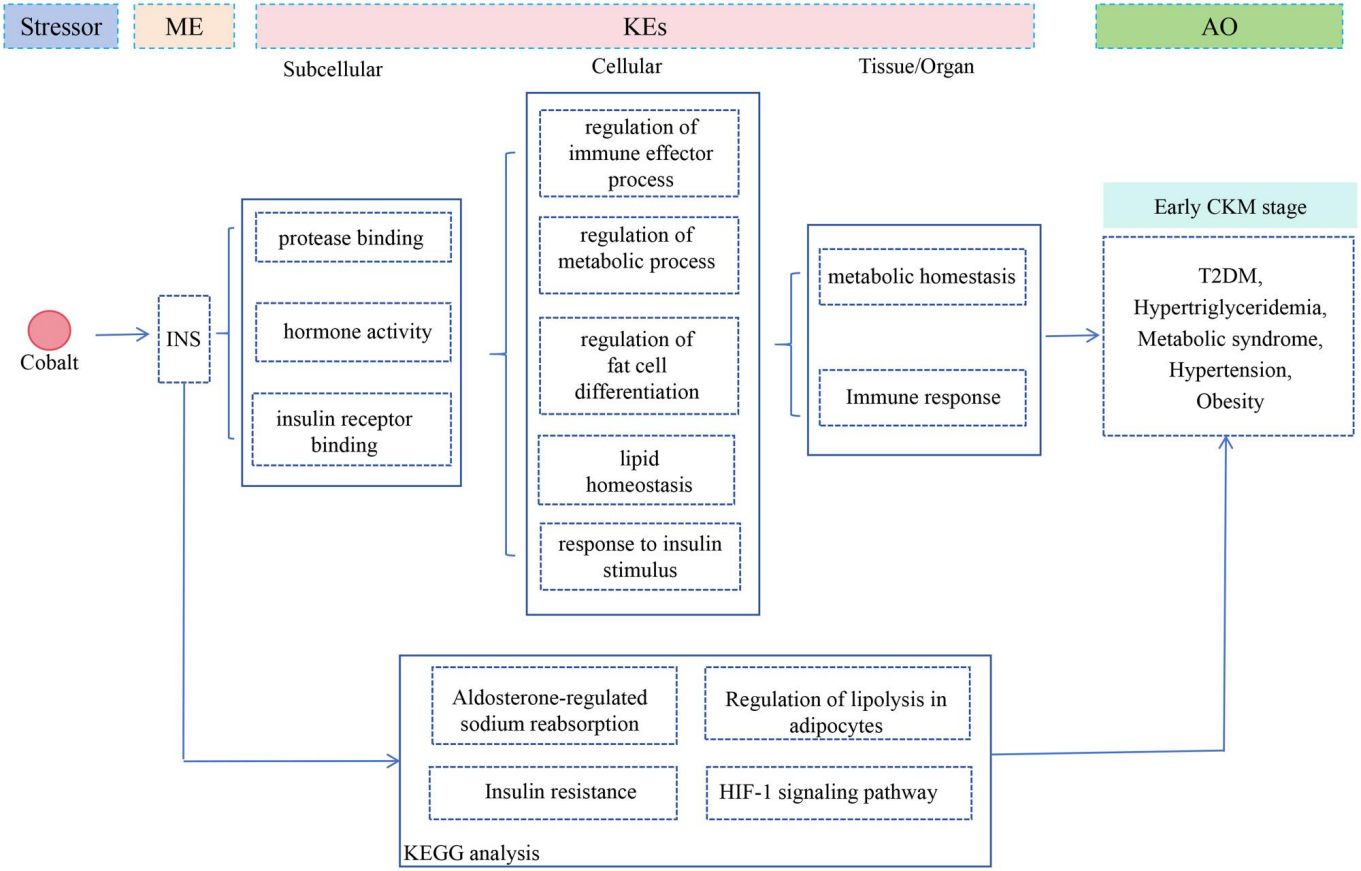
The network of the cobalt to the early CKM.

**Fig. 7.**
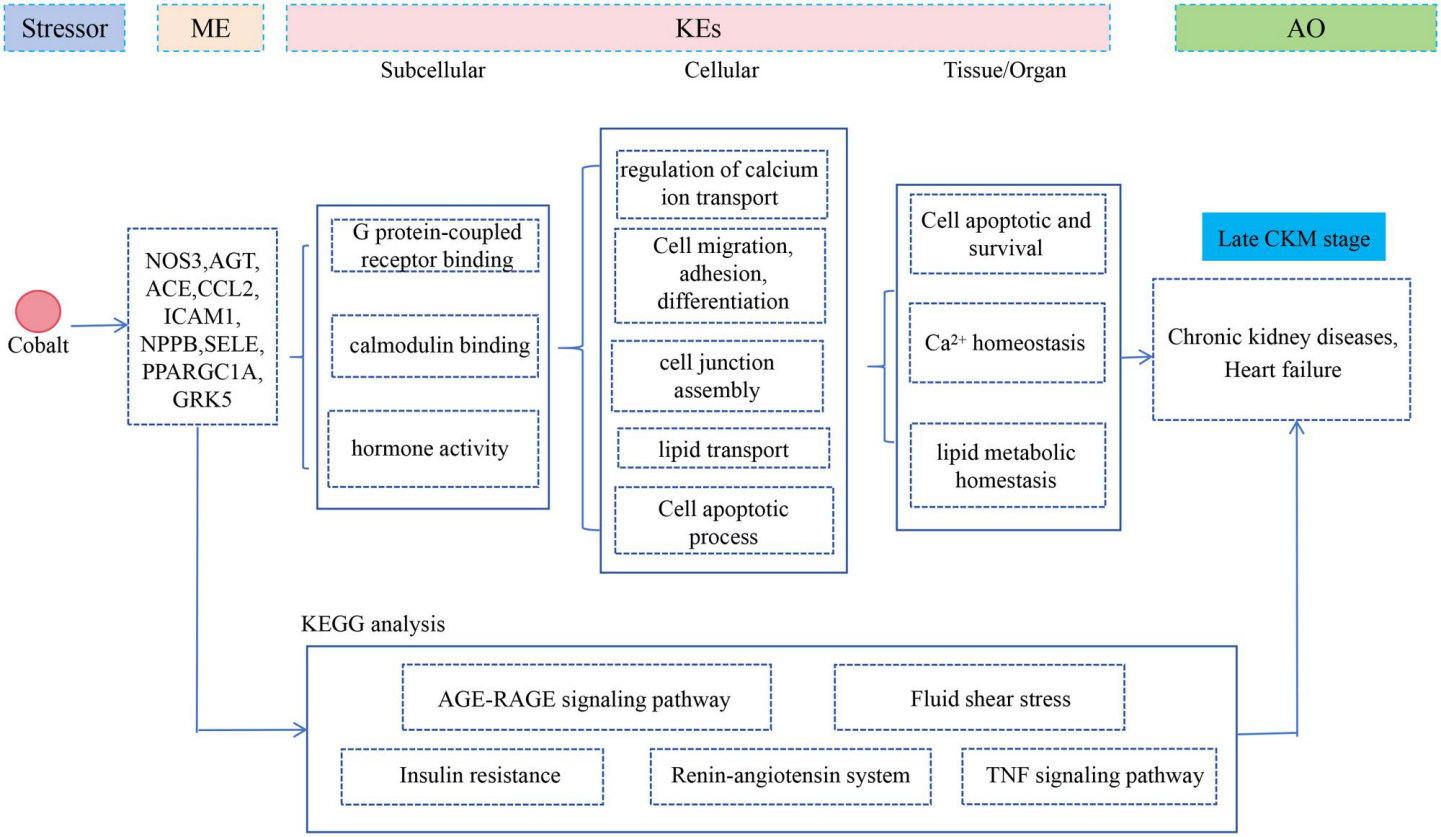
The network of the cobalt to the late CKM.

Through literature review combined with manual curation, we constructed the AOP framework of the early and late CKM, respectively. In the early CKM (Fig. 6), GO functions were categorized into subcellular levels (protease binding, hormone activity, INS receptor binding), cellular levels (regulation of immune effector processes, metabolic process regulation, adipose cell differentiation regulation, lipid homeostasis, INS stimulus–response), and organ levels (metabolic homeostasis, immune response), involving pathways such as aldosterone-regulated sodium reabsorption, regulation of lipolysis in adipocytes, INS resistance, and the Hypoxia-inducible factor (HIF-1) signaling pathway. For the late-stage CKM-associated AOP (Fig. 7), the phenotypes were classified into subcellular levels (G protein-coupled receptor binding, hormone activity, calmodulin binding), cellular levels (calcium ion transport regulation, cell migration, adhesion, differentiation, cell junction assembly, lipid transport, cell apoptotic process), and organ levels (cell apoptosis–survival balance, Ca2+ homeostasis, lipid metabolic homeostasis), involving pathways including the AGE-RAGE signaling pathway, fluid shear stress-mediated pathways, INS resistance, renin–angiotensin system, and the TNF signaling pathway.

## Discussion

A number of studies have indicated the association between metal exposure and cardiovascular disorders, including high blood pressure, arrhythmia, and atherosclerosis (Pan et al. 2024). One study based on the NHANES population reported that long-term exposure to a metal mixture was linked with obesity (Wang et al. 2018). A prospective study showed that exposure to 7 metals has a positive relationship with type 2 diabetes (Liu et al. 2024). Based on these findings, our study presents a pioneering evaluation of the progression of CKM syndrome from the perspective of mixture exposure. We employ multiple methodologies to unravel the intricate interactions among various metals. By categorizing CKM syndrome into early and advanced stages and using creatinine-adjusted urinary metal concentrations, our research offers unprecedented insights into the dose–response dynamics associated with metal exposure. This insight not only deepens our comprehension of the complex effects of metal mixtures on CKM syndrome but also highlights the necessity of accounting for exposure patterns in health risk assessments.

In our study, the initial correlation analyses revealed significant inter-metal correlations, after which the WQS model modeling demonstrated a positive relationship between mixed metal exposure and CKM syndrome, with uranium, tin, cadmium, lead, cesium, and cobalt exhibiting the highest weights. This ensemble approach effectively addresses multicollinearity among metals; besides, this implies that metal coexistence poses a potential challenge in facing the CKM syndrome progression. Subsequent ridge regression analyses for individual metal effects identified cadmium and cobalt as having statistically significant positive associations with CKM syndrome, partially consistent with WQS findings, suggesting their pivotal roles in disease progression. SHAP value interpretation further highlighted cobalt, cadmium, lead, and uranium as key drivers of CKM syndrome pathogenesis, providing machine learning validation of parametric models. In the polynomial regression analyses, the nonlinear associations were observed for barium, whereas cobalt exhibited a linear dose–response pattern, suggesting the absence of safe exposure thresholds within the studied ranges.

It is believed that the progression of CKM syndrome involves multiple pathological mechanisms, including inflammation, metabolic disruption, and INS resistance (Larkin 2023; Gao et al. 2024). Metals are naturally occurring elements globally that could intake by body and exert toxicity. Epidemiology research revealed that metal exposure is also connected with traditional MetS (Choi et al. 2023). Animal studies also showed that exposure to cadmium for 45 days leads to the metabolic disorders, Reactive Oxygen Species (ROS), and liver and kidney toxicity (Singh et al. 2012). Pregnant ICR mice exposed to lead could induce cardiotoxicity in offspring mice, with mitochondrial injury involved (Liu et al. 2023). Exposure to cadmium for 24 weeks could induce higher blood glucose with ROS activation in rats (Chen et al. 2024). One study reported that barium has the potential for carcinogenic toxicity (Kato et al. 2020), and another study also revealed that chronic exposure to high levels of barium could injure the kidney and cardiovascular function (Peana et al. 2021). Aligned with prior research, a meta-analysis showed that cadmium is related to the increasing risks of CVD (Verzelloni et al. 2024). Multiple metals, including Pb, Cd, Hg, and As, were the predominant risk factors for the workers’ kidney injury (Wang et al. 2024). Another study revealed a significant positive association between urinary cadmium concentrations and heart failure risk among non-smokers (Sears et al. 2022). Chronic exposure to cadmium leads to the progression of T2DM (Xiao et al. 2021). Elevated urinary cadmium levels are linked to an increased risk of MetS, particularly within the smoking population (Noor et al. 2018). Certain evidence revealed that urinary tin may inhibit the release of INS, which indicates its contribution to diabetes (Liu et al. 2018). Overall, our results indicated that metal exposure influences the progression of CKM.

Specifically, in our study, cobalt showed a consistently robust relationship with CKM syndrome, which demonstrated the independent linear relationship risk factor in the progression of CKM syndrome. Cobalt is a necessary element for the human body, and there may be no toxicity under physiology-relevant concentrations. However, excessive cobalt could be toxic to the body. Recently, a large population study revealed that urine cobalt is positive with CAC progression (McGraw et al. 2024). The potential toxicity mechanism is related to ROS production, mitochondrial injury, and DNA injury (Leyssens et al. 2017). Besides, we constructed a preliminary AOP framework to screen the potential mechanism in cobalt related the CKM. Notably, in the early-stage CKM, we noticed that metabolic homeostasis and immune response may exert certain effects in the progress of early CKM, whereas stepping into late CKM, cobalt may influence the cell apoptotsis and survival, Ca^2+^ homeostasis, and lipid metabolic homeostasis to the late CKM. Cobalt is a major raw material for new energy and is ubiquitously present in the environment. Therefore, it is necessary to focus on the effect of cobalt on the CKM syndrome in the future.

In conclusion, our study analyzed the relationship between metal exposure and CKM syndrome based on multiple models. Considering the linear relationship between cobalt and CKM syndrome, we also provide the AOP framework to understand the mechanism of cobalt related to CKM, highlighting critical implications for intervention policies.

## Supplementary Material

kfaf168_Supplementary_Data
