# Supplementary material for: Cobalt mediates stage-specific toxicity of metal mixtures in cardiovascular–kidney–metabolic syndrome
Source: Toxicol Sci. 2025 Dec 12;209(2):kfaf168. doi: 10.1093/toxsci/kfaf168 (PMC13117609; doi:10.1093/toxsci/kfaf168)
Supplement: kfaf168_Supplementary_Data [file kfaf168_supplementary_data.docx]

**Cobalt Mediates Stage-Specific Toxicity of Metal Mixtures in Cardiovascular-Kidney-Metabolic Syndrome**

Wei Zhang^1,2#^, GuangYu Jiang^1#^, Ziyan Liu^1^, LianRui Duan^1^, JiaYi Liang^1^, Ziyan Wang^1^, Huiwen Kang^1^, Danyang Huang^1^, Ai Gao^1,2*^

1 Department of Occupational Health and Environmental Health, School of Public Health, Capital Medical University, Beijing 100069, China

2 Beijing Key Laboratory of environment and aging, Capital Medical University, Beijing 100069, China

#These authors contributed equally to this study

*Corresponding author: Prof. Ai Gao

Address: Department of Occupational Health and Environmental Health, School of Public Health, Capital Medical University, 10 Xitoutiao, You An Men, Beijing 100069, China

Tel: +86–010–83911509

E-mail: gaoai428@ccmu.edu.cn (A. Gao)

**Methods**

**2.3. Cardiovascular-Kidney-Metabolic Syndrome definition**

The classification framework for cardiometabolic-kidney (CKM) disease progression (stratified into phases 0 through 4) was established through the adaptation of existing diagnostic protocols (Aggarwal et al. 2024), with methodological adaptations for NHANES dataset compatibility. Phase 0 comprised individuals maintaining anthropometric parameters within normative ranges (BMI 18.5-24.9 kg/m² combined with waist circumference <88 cm [female] or < 102 cm [male]) without exhibiting subsequent phase-defining characteristics. Phase 1 encompassed subjects demonstrating either elevated adiposity indicators (BMI ≥ 25 kg/m² or abdominal obesity thresholds ≥88 cm [female] ≥ 102 cm [male]) or impaired glucose regulation (prediabetic status). The diagnosis of hypertension and diabetes was based on self-reports in the questionnaire.

Progression to Phase 2 required documentation of either: (1) clinically significant metabolic dysregulation (fasting triglycerides ≥ 150 mg/dL, hypertension diagnosis, confirmed diabetes, or metabolic syndrome criteria fulfillment) or (2) moderate-severe chronic kidney disease as per international nephrology guidelines (Group 2024). Phase 3 designation applied to individuals presenting with either end-stage renal impairment or elevated cardiovascular risk profiles (10-year atherosclerotic cardiovascular disease [ASCVD] probability ≥ 20% calculated via AHA PREVENT risk modeling (Khan et al. 2024), validated for adults 30-79 years). Phase 4 was reserved for subjects with documented cardiovascular pathology (including ischemic heart disease, acute coronary events, cardiac insufficiency, or cerebrovascular accidents).

Renal function assessment incorporated the estimated glomerular filtration rate (eGFR) derived from CKD-EPI creatinine formulae combined with urinary albumin excretion quantification. Analytical models operationalized CKM syndrome staging both as categorical (ordinal 0-4 scale) and dichotomized variables, the latter distinguishing early-phase (stages 0-2) from advanced-phase (stages 3-4) clusters in subsequent multivariable analyses.

**2.4. Concomitant variable**

The adjusted covariates included sociodemographic data (age, gender, ethnicity, education level, annual household income, marital status, body mass index (BMI), physical activity, smoking status, alcohol consumption, diabetes, and hypertension).

Age is divided into three stages: young (20-35), middle (36-59), and old (>=60). BMI is calculated with body weight (kg) / height (m^2^) and divided into three stages, low (< 18.5 kg/m^2^), normal (18.5-24.9 kg/m^2^), overweight (≥25.0 kg/m^2^), and obese (≥30.0 kg/m^2^). Based on the questionnaire data, smokers were defined as those who used any tobacco product for the last 5 days, alcohol consumers were defined as those who consumed at least 12 alcoholic beverages per year (Huang et al. 2024), and physical activity refers to does your work involve a moderate-intensity activity that causes small increases in breathing or heart rate such as brisk walking or carrying light loads for at least 10 minutes continuously, Lancet reported that even intermittent non-exercise physical activity is beneficial for reducing the major adverse cardiovascular events (Ahmadi et al. 2023). Diabetes diagnosis was determined as fasting blood glucose ≥ 7.0 mmol/L or based on the questionnaire data. A high blood pressure diagnosis is determined based on the answer to the question (whether a doctor or other health professional has ever told you that you have high blood pressure).

**2.5. Data analysis**

**2.5.1. Generalized Linear Models**

A generalized linear model is constructed by using the multi-variable adjustment strategy in stages. Unadjusted Model: only metal variables are included; Demographics Adjusted: gender, education, and ethnic covariates are added to the rough model; Fully Adjusted Model: age, poverty, marital status, BMI, smoking, alcohol consumption, hypertension, physical activity, and diabetes were further included. All models are fitted by the svyglm function, and the quasibinomial family is used to deal with the over-discretization problem. The estimation of the model parameters is based on the adjustment of the design effects, and the T-distribution method is used to calculate the confidence interval. The ggplot2 package was used to generate hierarchical forest maps to visualize the results, including the standardized Beta coefficient (Beta), 95% confidence interval (95% CI), and P-value.

**2.5.2. WQS (Weighted Quantile Sum)**

WQS serves as a statistical model for multiple regression analysis in high-dimensional datasets. It constructs a weighted index to evaluate the association with the outcome variable and estimates the collective impact of all exposure variables on the outcome.

The WQS model was formulated as:

logit(P(Y=1)) = β_0_+β_WQS_⋅WQS+ $\sum_{k=1}^{p} \gamma_{k}C_{k}$

Where Y represents the binary outcome of CKM syndrome progression, WQS denotes the weighted index of chemical exposures, and C_k_​ corresponds to adjusted covariates including gender, age, education, marital status, ethnicity, physical activity, hypertension, diabetes, BMI, alcohol, and tobacco. In this study, the data was divided into a 40% training set and a 60% verification set, 1000 bootstrap iterations were selected by self-sampling, and one seed was chosen to guarantee reproducibility.

**2.5.3. Ridge Regression**

The penalized regression with ridge regularization to address multicollinearity in high-dimensional exposure data. Continuous toxicant concentrations underwent z-score standardization while categorical covariates were encoded through model matrix expansion. A binomial family ridge regression was implemented via 10-fold cross-validation using the glmnet package, with optimal regularization parameter (λ=0.033) selected by minimum cross-validated deviance. To enhance result reliability, a stability selection procedure was conducted with 100 bootstrap resamples, retaining variables with ≥60% selection frequency. Coefficient estimates were extracted at the optimal λ, with stability metrics calculated as empirical inclusion probabilities across resamples. Final results visualization utilized ggplot2 for creating coefficient plots. The computational workflow included seed fixation (seed=123) for reproducibility and explicit missing value handling during coefficient extraction.

**2.5.4. Polynomial Regression**

Binomial logistic regression with quadratic terms to evaluate dose-response relationships between 13 metal exposures and late-stage CKM risk, adjusting for age, sex, ethnicity, diabetes, and BMI. Inflection points (log₁₀-transformed and original concentrations, and corresponding probabilities) calculated for significant quadratic terms (p < 0.05). Analyses included visualization of predicted probabilities and 95% confidence intervals across observed metal concentrations (log₁₀ scale), incorporating smoothed curves and raw data jitter plots. Results were synthesized to report regression coefficients, significance, AIC, deviance, and critical points (inflection or threshold) where applicable, providing a comprehensive assessment of linear and nonlinear associations.

**2.5.5. K-means Clustering and t-SNE Visualization**

The analytical framework integrated machine learning interpretability techniques with cluster-based stratification, commencing with z-score standardization of exposure variables followed by k-means clustering (k=2, nstart=25) validated through silhouette coefficient analysis (mean width=0.42). Dimensionality reduction employed t-SNE visualization (perplexity=30, theta=0.5, 1000 iterations) for cluster pattern verification. Gradient-boosted modeling utilized XGBoost with fixed hyperparameters (learning rate=0.1, 65 boosting rounds) and stratified SHAP value computation through the shapviz package, implementing Python interoperability via reticulate with 1,816 background samples. Comparative analysis between exposure clusters incorporated bootstrap-consistent model rebuilding (seed=3653) and differential SHAP effect quantification through mean absolute value aggregation. Technical safeguards included automated one-hot encoding for categorical variables, column name consistency verification during SHAP object construction, and explicit missing value handling in coefficient extraction. Computational reproducibility was ensured through systematic seed setting (123 for clustering, 3653 for XGBoost) and version-controlled environment management (conda environment "r-reticulate").

**Result**

**Fig S1. The workflow of the AOP construction**


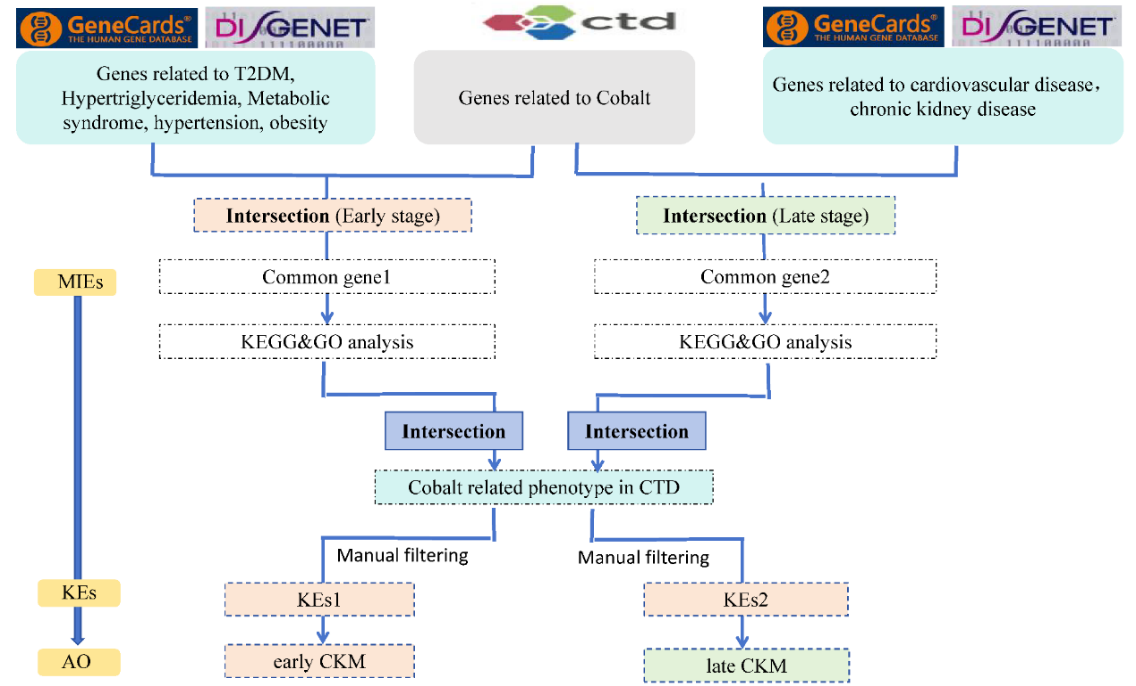


**Fig S2. The heatmap showed the relationship between the metals and the CKM syndrome stage after adjusting covariates**


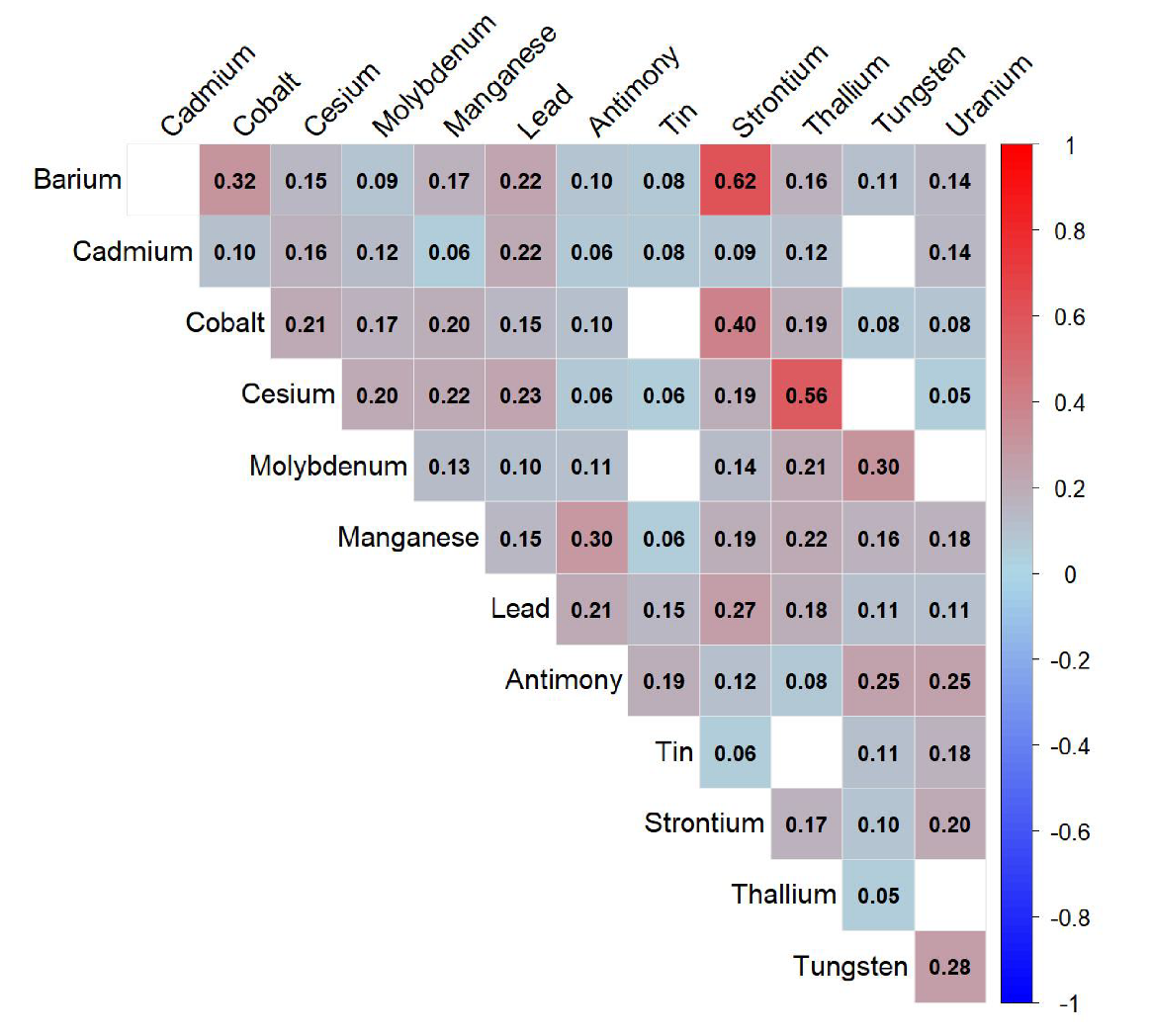


**Fig S3. Feature importance plot was used to show the contribution of covariates**


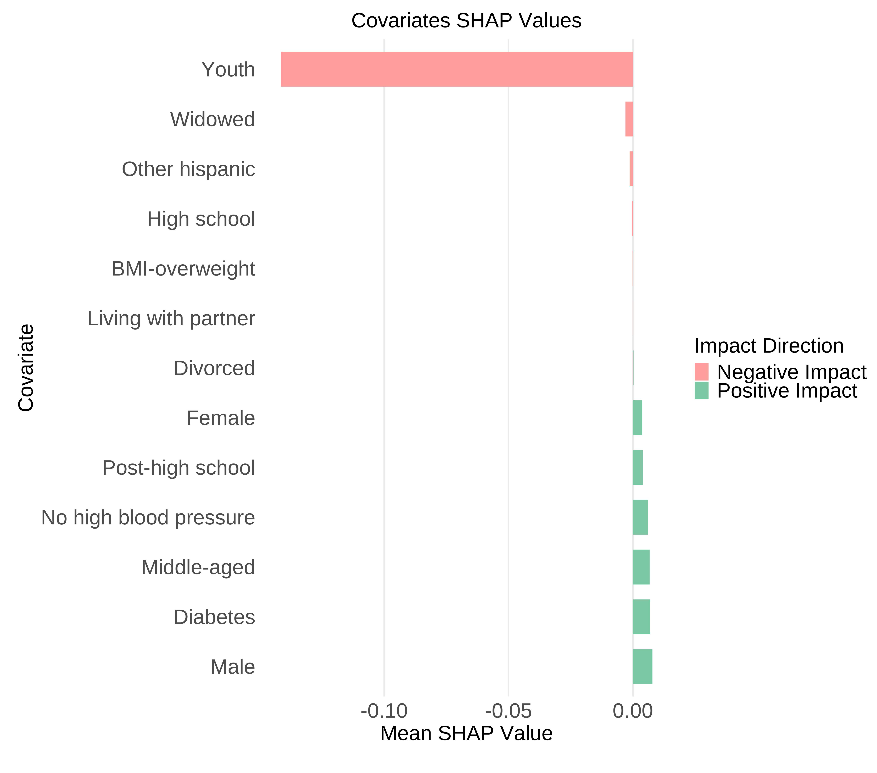


**Fig S4. The standardized weight values of each metal in the WQS, GLM, and ridge regression models were summarized by using radar plots**


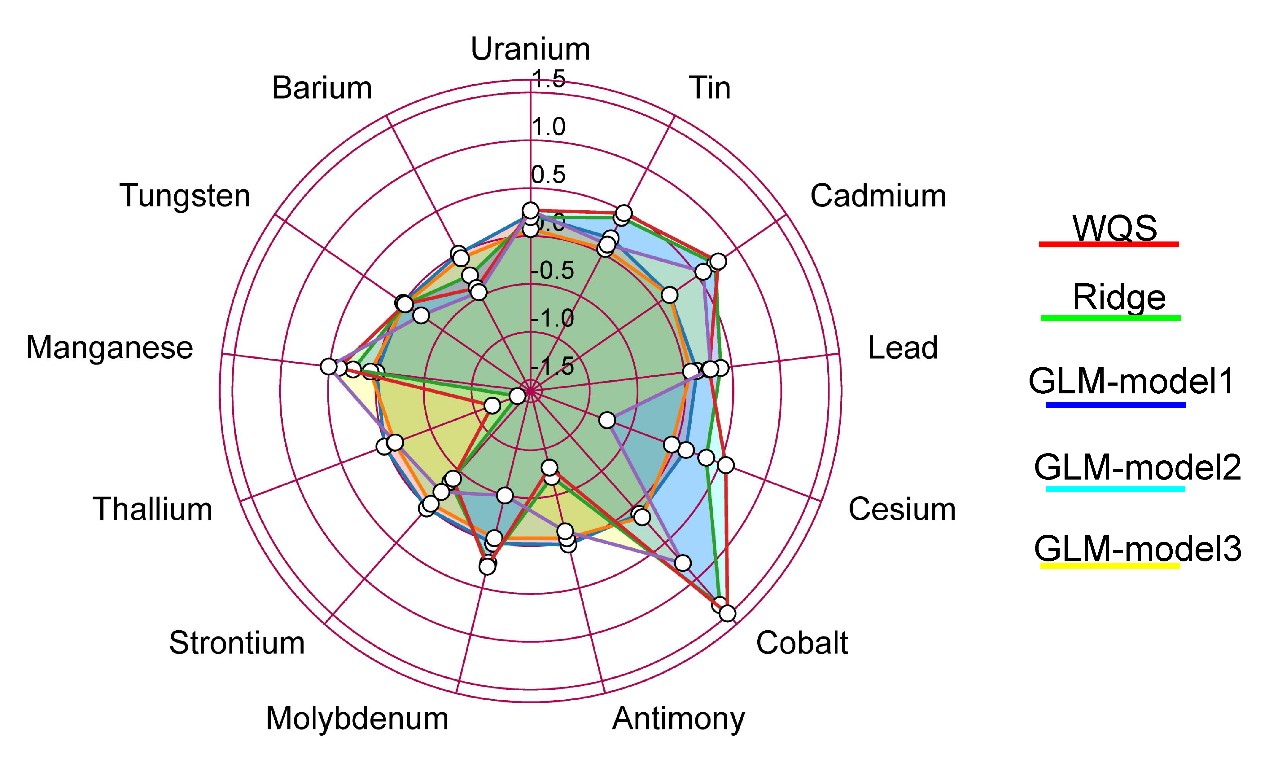


**Table S1. Stratified analysis of the association between metal exposure and CKM risk by smoking status**

| Metals | Smoking  Group | Beta | SE | OR | CI_95 | P_Value |
| --- | --- | --- | --- | --- | --- | --- |
| Antimony | No | -33.533 | 103.223 | 2.73e-15 | 3.73e-103-2.00e+73 | 0.7514 |
| Antimony | Yes | -687.571 | 708.645 | 2.47e-299 | 0.00e+00-4.00e+304 | 0.3573 |
| Barium | No | -21.428 | 15.648 | 4.94e-10 | 2.37e-23-1.03e+04 | 0.1982 |
| Barium | Yes | -69.301 | 77.03 | 8.00e-31 | 2.16e-96-2.97e+35 | 0.3917 |
| Cadmium | No | 186.954 | 79.099 | 1.56e+81 | 7.29e+13-3.33e+148 | 0.0376 |
| Cadmium | Yes | -87.456 | 143.96 | 1.04e-38 | 3.00e-161-3.62e+84 | 0.5585 |
| Cesium | No | -38.638 | 18.176 | 1.66e-17 | 5.60e-33-0.05 | 0.057 |
| Cesium | Yes | 23.913 | 46.636 | 2.43e+10 | 4.88e-30-1.21e+50 | 0.6205 |
| Cobalt | No | 48.518 | 24.388 | 1.18e+21 | 2.05-6.76e+41 | 0.0721 |
| Cobalt | Yes | 143.826 | 144.544 | 2.90e+62 | 2.66e-61-3.17e+185 | 0.3457 |
| Lead | No | 19.668 | 17.202 | 3.48e+08 | 7.93e-07-1.53e+23 | 0.2772 |
| Lead | Yes | 58.538 | 52.42 | 2.65e+25 | 6.33e-20-1.11e+70 | 0.293 |
| Manganese | No | 80.982 | 25.909 | 1.48e+35 | 1.30e+13-1.68e+57 | 0.0097 |
| Manganese | Yes | -1296.547 | 474.527 | 0.00e+00 | 0.00e+00-6.95e-160 | 0.0231 |
| Molybdenum | No | -3.467 | 2.105 | 0.03 | 5.04e-04-1.93 | 0.1278 |
| Molybdenum | Yes | -0.561 | 5.234 | 0.57 | 2.00e-05-1.63e+04 | 0.917 |
| Strontium | No | -0.975 | 1.452 | 0.38 | 0.02-6.49 | 0.5158 |
| Strontium | Yes | 1 | 2.041 | 2.72 | 0.05-148.56 | 0.6359 |
| Thallium | No | -8.562 | 435.442 | 1.91e-04 | 0.00e+00-Inf | 0.9847 |
| Thallium | Yes | 402.881 | 820.519 | 9.31e+174 | 0.00e+00-Inf | 0.6352 |
| Tin | No | -1.611 | 7.651 | 0.2 | 6.14e-08-6.50e+05 | 0.8371 |
| Tin | Yes | 12.327 | 7.859 | 2.26e+05 | 0.05-1.11e+12 | 0.1512 |
| Tungsten | No | -103.857 | 88.279 | 7.86e-46 | 5.63e-121-1.10e+30 | 0.2642 |
| Tungsten | Yes | 44.798 | 728.108 | 2.86e+19 | 0.00e+00-Inf | 0.9523 |
| Uranium | No | 856.104 | 917.109 | Inf | 0.00e+00-Inf | 0.3706 |
| Uranium | Yes | 177.274 | 2523.76 | 9.75e+76 | 0.00e+00-Inf | 0.9455 |

| **Table S2. The genes abundance in heart and kidney tissues, compared with other tissues** | | | | | |
| --- | --- | --- | --- | --- | --- |
| Genes | Median TPM | |  | Rank | |
|  | Kidney | Heart |  | Kidney | Heart |
| NOS3 | 23.4 | 19.9 |  | 2 | 3 |
| AGT | 149.6 | 51 |  | 12 | 7 |
| ACE | 10.4 | 10.1 |  | 13 | 14 |
| CCL2 | 222.6 | 295.6 |  | 6 | 4 |
| ICAM1 | 42.5 | 37.8 |  | 9 | 10 |
| NPPB | 0 | 4968.9 |  | 0 | 1 |
| SELE | 7.7 | 7.4 |  | 15 | 14 |
| PPARGC1A | 28 | 35 |  | 7 | 4 |
| GRK5 | 13.8 | 47.3 |  | 18 | 2 |

| **Table S3. The intersection GO phenotype of the early CKM between the GO analysis based on the INS and the phenotypes related to the cobalt in the CTD** | | | | | |
| --- | --- | --- | --- | --- | --- |
| Common ID | Ontology | GO Term Name | Rich  Factor | Fold  Enrichment | pvalue |
| GO:2001233 | Biological Process | regulation of apoptotic signaling pathway | 0.003 | 47.697 | 0.021 |
| GO:0043434 | Biological Process | response to peptide hormone | 0.002 | 43.123 | 0.023 |
| GO:0042060 | Biological Process | wound healing | 0.002 | 43.926 | 0.023 |
| GO:0019693 | Biological Process | ribose phosphate metabolic process | 0.002 | 39.350 | 0.025 |
| GO:1901653 | Biological Process | cellular response to peptide | 0.003 | 50.234 | 0.020 |
| GO:0009259 | Biological Process | ribonucleotide metabolic process | 0.002 | 40.532 | 0.025 |
| GO:0097193 | Biological Process | intrinsic apoptotic signaling pathway | 0.003 | 59.210 | 0.017 |
| GO:0009150 | Biological Process | purine ribonucleotide metabolic process | 0.002 | 42.350 | 0.024 |
| GO:2001234 | Biological Process | negative regulation of apoptotic signaling pathway | 0.004 | 77.410 | 0.013 |
| GO:0016049 | Biological Process | cell growth | 0.002 | 38.081 | 0.026 |
| GO:0042176 | Biological Process | regulation of protein catabolic process | 0.003 | 52.177 | 0.019 |
| GO:0048285 | Biological Process | organelle fission | 0.002 | 38.312 | 0.026 |
| GO:0071375 | Biological Process | cellular response to peptide hormone stimulus | 0.003 | 60.733 | 0.016 |
| GO:0000280 | Biological Process | nuclear division | 0.002 | 42.350 | 0.024 |
| GO:0051223 | Biological Process | regulation of protein transport | 0.002 | 42.927 | 0.023 |
| GO:0032102 | Biological Process | negative regulation of response to external stimulus | 0.002 | 41.421 | 0.024 |
| GO:0072593 | Biological Process | reactive oxygen species metabolic process | 0.004 | 79.361 | 0.013 |
| GO:0001819 | Biological Process | positive regulation of cytokine production | 0.002 | 37.776 | 0.026 |
| GO:0001558 | Biological Process | regulation of cell growth | 0.002 | 45.187 | 0.022 |
| GO:0140014 | Biological Process | mitotic nuclear division | 0.004 | 68.188 | 0.015 |
| GO:0045088 | Biological Process | regulation of innate immune response | 0.002 | 42.927 | 0.023 |
| GO:0043410 | Biological Process | positive regulation of MAPK cascade | 0.002 | 39.764 | 0.025 |
| GO:0062012 | Biological Process | regulation of small molecule metabolic process | 0.003 | 57.063 | 0.018 |
| GO:1903829 | Biological Process | positive regulation of protein localization | 0.002 | 39.106 | 0.026 |
| GO:0005996 | Biological Process | monosaccharide metabolic process | 0.004 | 74.952 | 0.013 |
| GO:0043491 | Biological Process | protein kinase B signaling | 0.003 | 63.171 | 0.016 |
| GO:0006520 | Biological Process | amino acid metabolic process | 0.003 | 64.027 | 0.016 |
| GO:0003018 | Biological Process | vascular process in circulatory system | 0.004 | 68.684 | 0.015 |
| GO:0032868 | Biological Process | response to insulin | 0.004 | 70.216 | 0.014 |
| GO:0009895 | Biological Process | negative regulation of catabolic process | 0.003 | 53.507 | 0.019 |
| GO:0019318 | Biological Process | hexose metabolic process | 0.004 | 81.414 | 0.012 |
| GO:0019216 | Biological Process | regulation of lipid metabolic process | 0.003 | 57.939 | 0.017 |
| GO:0009141 | Biological Process | nucleoside triphosphate metabolic process | 0.004 | 71.008 | 0.014 |
| GO:0051896 | Biological Process | regulation of protein kinase B signaling | 0.004 | 74.656 | 0.013 |
| GO:0050727 | Biological Process | regulation of inflammatory response | 0.002 | 44.442 | 0.023 |
| GO:0045787 | Biological Process | positive regulation of cell cycle | 0.003 | 56.892 | 0.018 |
| GO:0006631 | Biological Process | fatty acid metabolic process | 0.002 | 47.102 | 0.021 |
| GO:0045859 | Biological Process | regulation of protein kinase activity | 0.002 | 40.187 | 0.025 |
| GO:2001242 | Biological Process | regulation of intrinsic apoptotic signaling pathway | 0.005 | 99.411 | 0.010 |
| GO:1990778 | Biological Process | protein localization to cell periphery | 0.003 | 53.056 | 0.019 |
| GO:0015980 | Biological Process | energy derivation by oxidation of organic compounds | 0.003 | 55.882 | 0.018 |
| GO:0031331 | Biological Process | positive regulation of cellular catabolic process | 0.002 | 43.722 | 0.023 |
| GO:0034504 | Biological Process | protein localization to nucleus | 0.003 | 60.733 | 0.016 |
| GO:0009144 | Biological Process | purine nucleoside triphosphate metabolic process | 0.004 | 75.552 | 0.013 |
| GO:0009199 | Biological Process | ribonucleoside triphosphate metabolic process | 0.004 | 75.251 | 0.013 |
| GO:0032869 | Biological Process | cellular response to insulin stimulus | 0.005 | 91.689 | 0.011 |
| GO:0009205 | Biological Process | purine ribonucleoside triphosphate metabolic process | 0.004 | 77.410 | 0.013 |
| GO:0010975 | Biological Process | regulation of neuron projection development | 0.002 | 41.061 | 0.024 |
| GO:0002697 | Biological Process | regulation of immune effector process | 0.003 | 49.060 | 0.020 |
| GO:0051051 | Biological Process | negative regulation of transport | 0.002 | 40.883 | 0.024 |
| GO:0046034 | Biological Process | ATP metabolic process | 0.005 | 85.855 | 0.012 |
| GO:0006006 | Biological Process | glucose metabolic process | 0.005 | 98.375 | 0.010 |
| GO:0072659 | Biological Process | protein localization to plasma membrane | 0.003 | 64.464 | 0.016 |
| GO:2000377 | Biological Process | regulation of reactive oxygen species metabolic process | 0.007 | 126.765 | 0.008 |
| GO:0051222 | Biological Process | positive regulation of protein transport | 0.004 | 75.251 | 0.013 |
| GO:0031348 | Biological Process | negative regulation of defense response | 0.003 | 64.907 | 0.015 |
| GO:0090068 | Biological Process | positive regulation of cell cycle process | 0.004 | 71.817 | 0.014 |
| GO:1904951 | Biological Process | positive regulation of establishment of protein localization | 0.003 | 57.939 | 0.017 |
| GO:0031346 | Biological Process | positive regulation of cell projection organization | 0.003 | 52.467 | 0.019 |
| GO:0016042 | Biological Process | lipid catabolic process | 0.003 | 55.553 | 0.018 |
| GO:0034762 | Biological Process | regulation of transmembrane transport | 0.002 | 38.626 | 0.026 |
| GO:0031330 | Biological Process | negative regulation of cellular catabolic process | 0.004 | 84.321 | 0.012 |
| GO:0051897 | Biological Process | positive regulation of protein kinase B signaling | 0.006 | 110.456 | 0.009 |
| GO:0016052 | Biological Process | carbohydrate catabolic process | 0.006 | 119.544 | 0.008 |
| GO:0071692 | Biological Process | protein localization to extracellular region | 0.003 | 49.836 | 0.020 |
| GO:0035592 | Biological Process | establishment of protein localization to extracellular region | 0.003 | 50.911 | 0.020 |
| GO:0043086 | Biological Process | negative regulation of catalytic activity | 0.002 | 42.067 | 0.024 |
| GO:0009306 | Biological Process | protein secretion | 0.003 | 51.049 | 0.020 |
| GO:2001243 | Biological Process | negative regulation of intrinsic apoptotic signaling pathway | 0.009 | 165.684 | 0.006 |
| GO:0051347 | Biological Process | positive regulation of transferase activity | 0.003 | 50.234 | 0.020 |
| GO:0042180 | Biological Process | cellular ketone metabolic process | 0.004 | 83.207 | 0.012 |
| GO:0051047 | Biological Process | positive regulation of secretion | 0.003 | 60.538 | 0.017 |
| GO:0045444 | Biological Process | fat cell differentiation | 0.004 | 76.161 | 0.013 |
| GO:0050808 | Biological Process | synapse organization | 0.002 | 38.312 | 0.026 |
| GO:0023061 | Biological Process | signal release | 0.002 | 38.784 | 0.026 |
| GO:0009132 | Biological Process | nucleoside diphosphate metabolic process | 0.008 | 148.724 | 0.007 |
| GO:0042593 | Biological Process | glucose homeostasis | 0.004 | 73.209 | 0.014 |
| GO:0009185 | Biological Process | ribonucleoside diphosphate metabolic process | 0.008 | 157.400 | 0.006 |
| GO:0033500 | Biological Process | carbohydrate homeostasis | 0.004 | 72.927 | 0.014 |
| GO:0046890 | Biological Process | regulation of lipid biosynthetic process | 0.005 | 101.005 | 0.010 |
| GO:0006109 | Biological Process | regulation of carbohydrate metabolic process | 0.005 | 103.780 | 0.010 |
| GO:1903532 | Biological Process | positive regulation of secretion by cell | 0.003 | 65.583 | 0.015 |
| GO:0016051 | Biological Process | carbohydrate biosynthetic process | 0.005 | 91.689 | 0.011 |
| GO:0099177 | Biological Process | regulation of trans-synaptic signaling | 0.002 | 38.235 | 0.026 |
| GO:0008286 | Biological Process | insulin receptor signaling pathway | 0.008 | 149.905 | 0.007 |
| GO:0050804 | Biological Process | modulation of chemical synaptic transmission | 0.002 | 38.312 | 0.026 |
| GO:0009135 | Biological Process | purine nucleoside diphosphate metabolic process | 0.009 | 171.709 | 0.006 |
| GO:0009179 | Biological Process | purine ribonucleoside diphosphate metabolic process | 0.009 | 171.709 | 0.006 |
| GO:0043467 | Biological Process | regulation of generation of precursor metabolites and energy | 0.007 | 134.914 | 0.007 |
| GO:1900180 | Biological Process | regulation of protein localization to nucleus | 0.007 | 131.167 | 0.008 |
| GO:0046031 | Biological Process | ADP metabolic process | 0.010 | 192.735 | 0.005 |
| GO:0009914 | Biological Process | hormone transport | 0.003 | 58.658 | 0.017 |
| GO:0097746 | Biological Process | blood vessel diameter maintenance | 0.007 | 124.263 | 0.008 |
| GO:0035296 | Biological Process | regulation of tube diameter | 0.007 | 124.263 | 0.008 |
| GO:0035150 | Biological Process | regulation of tube size | 0.007 | 123.451 | 0.008 |
| GO:0050708 | Biological Process | regulation of protein secretion | 0.004 | 70.216 | 0.014 |
| GO:1905475 | Biological Process | regulation of protein localization to membrane | 0.006 | 104.933 | 0.010 |
| GO:0051090 | Biological Process | regulation of DNA-binding transcription factor activity | 0.003 | 49.575 | 0.020 |
| GO:0046879 | Biological Process | hormone secretion | 0.003 | 60.733 | 0.016 |
| GO:1903828 | Biological Process | negative regulation of protein localization | 0.005 | 87.444 | 0.011 |
| GO:0050803 | Biological Process | regulation of synapse structure or activity | 0.004 | 75.855 | 0.013 |
| GO:0006090 | Biological Process | pyruvate metabolic process | 0.008 | 160.068 | 0.006 |
| GO:0050807 | Biological Process | regulation of synapse organization | 0.004 | 77.728 | 0.013 |
| GO:0050777 | Biological Process | negative regulation of immune response | 0.005 | 97.361 | 0.010 |
| GO:0062013 | Biological Process | positive regulation of small molecule metabolic process | 0.007 | 133.014 | 0.008 |
| GO:0045834 | Biological Process | positive regulation of lipid metabolic process | 0.008 | 153.561 | 0.007 |
| GO:0033674 | Biological Process | positive regulation of kinase activity | 0.003 | 62.337 | 0.016 |
| GO:0045927 | Biological Process | positive regulation of growth | 0.004 | 73.209 | 0.014 |
| GO:0042886 | Biological Process | amide transport | 0.003 | 60.538 | 0.017 |
| GO:0006096 | Biological Process | glycolytic process | 0.011 | 209.867 | 0.005 |
| GO:0055088 | Biological Process | lipid homeostasis | 0.006 | 107.318 | 0.009 |
| GO:0045860 | Biological Process | positive regulation of protein kinase activity | 0.004 | 73.494 | 0.014 |
| GO:0099173 | Biological Process | postsynapse organization | 0.005 | 96.367 | 0.010 |
| GO:0051783 | Biological Process | regulation of nuclear division | 0.007 | 128.490 | 0.008 |
| GO:0046883 | Biological Process | regulation of hormone secretion | 0.004 | 74.362 | 0.013 |
| GO:0010565 | Biological Process | regulation of cellular ketone metabolic process | 0.007 | 131.167 | 0.008 |
| GO:0007088 | Biological Process | regulation of mitotic nuclear division | 0.008 | 160.068 | 0.006 |
| GO:0034764 | Biological Process | positive regulation of transmembrane transport | 0.005 | 88.676 | 0.011 |
| GO:0050890 | Biological Process | cognition | 0.003 | 58.658 | 0.017 |
| GO:0046631 | Biological Process | alpha-beta T cell activation | 0.006 | 104.354 | 0.010 |
| GO:0010906 | Biological Process | regulation of glucose metabolic process | 0.010 | 187.010 | 0.005 |
| GO:0008631 | Biological Process | intrinsic apoptotic signaling pathway in response to oxidative stress | 0.016 | 295.125 | 0.003 |
| GO:0046364 | Biological Process | monosaccharide biosynthetic process | 0.010 | 196.750 | 0.005 |
| GO:0015833 | Biological Process | peptide transport | 0.004 | 69.697 | 0.014 |
| GO:1900182 | Biological Process | positive regulation of protein localization to nucleus | 0.011 | 200.936 | 0.005 |
| GO:0045861 | Biological Process | negative regulation of proteolysis | 0.004 | 81.766 | 0.012 |
| GO:0002832 | Biological Process | negative regulation of response to biotic stimulus | 0.008 | 158.723 | 0.006 |
| GO:1904375 | Biological Process | regulation of protein localization to cell periphery | 0.007 | 137.869 | 0.007 |
| GO:0008643 | Biological Process | carbohydrate transport | 0.006 | 118.792 | 0.008 |
| GO:0002526 | Biological Process | acute inflammatory response | 0.009 | 173.284 | 0.006 |
| GO:0019319 | Biological Process | hexose biosynthetic process | 0.011 | 205.304 | 0.005 |
| GO:0034219 | Biological Process | carbohydrate transmembrane transport | 0.007 | 137.869 | 0.007 |
| GO:0099175 | Biological Process | regulation of postsynapse organization | 0.010 | 183.379 | 0.005 |
| GO:0006094 | Biological Process | gluconeogenesis | 0.011 | 212.225 | 0.005 |
| GO:1900076 | Biological Process | regulation of cellular response to insulin stimulus | 0.014 | 262.333 | 0.004 |
| GO:0015749 | Biological Process | monosaccharide transmembrane transport | 0.008 | 153.561 | 0.007 |
| GO:0072524 | Biological Process | pyridine-containing compound metabolic process | 0.006 | 111.106 | 0.009 |
| GO:0051091 | Biological Process | positive regulation of DNA-binding transcription factor activity | 0.004 | 82.122 | 0.012 |
| GO:1904659 | Biological Process | glucose transmembrane transport | 0.008 | 160.068 | 0.006 |
| GO:0050728 | Biological Process | negative regulation of inflammatory response | 0.005 | 93.970 | 0.011 |
| GO:0042177 | Biological Process | negative regulation of protein catabolic process | 0.009 | 165.684 | 0.006 |
| GO:0051048 | Biological Process | negative regulation of secretion | 0.006 | 109.179 | 0.009 |
| GO:0008645 | Biological Process | hexose transmembrane transport | 0.008 | 156.099 | 0.006 |
| GO:1901136 | Biological Process | carbohydrate derivative catabolic process | 0.004 | 69.187 | 0.014 |
| GO:1900542 | Biological Process | regulation of purine nucleotide metabolic process | 0.011 | 207.560 | 0.005 |
| GO:0010976 | Biological Process | positive regulation of neuron projection development | 0.006 | 117.317 | 0.009 |
| GO:0006140 | Biological Process | regulation of nucleotide metabolic process | 0.011 | 205.304 | 0.005 |
| GO:1902175 | Biological Process | regulation of oxidative stress-induced intrinsic apoptotic signaling pathway | 0.021 | 401.872 | 0.002 |
| GO:0002790 | Biological Process | peptide secretion | 0.004 | 76.470 | 0.013 |
| GO:0030307 | Biological Process | positive regulation of cell growth | 0.006 | 115.877 | 0.009 |
| GO:1903076 | Biological Process | regulation of protein localization to plasma membrane | 0.009 | 174.889 | 0.006 |
| GO:0046626 | Biological Process | regulation of insulin receptor signaling pathway | 0.014 | 269.829 | 0.004 |
| GO:0030072 | Biological Process | peptide hormone secretion | 0.004 | 78.050 | 0.013 |
| GO:0048167 | Biological Process | regulation of synaptic plasticity | 0.005 | 87.444 | 0.011 |
| GO:0046496 | Biological Process | nicotinamide nucleotide metabolic process | 0.006 | 115.171 | 0.009 |
| GO:0019362 | Biological Process | pyridine nucleotide metabolic process | 0.006 | 115.171 | 0.009 |
| GO:0005976 | Biological Process | polysaccharide metabolic process | 0.010 | 187.010 | 0.005 |
| GO:1904950 | Biological Process | negative regulation of establishment of protein localization | 0.008 | 142.015 | 0.007 |
| GO:0051224 | Biological Process | negative regulation of protein transport | 0.008 | 160.068 | 0.006 |
| GO:0009308 | Biological Process | amine metabolic process | 0.008 | 160.068 | 0.006 |
| GO:0046889 | Biological Process | positive regulation of lipid biosynthetic process | 0.011 | 214.636 | 0.005 |
| GO:0062014 | Biological Process | negative regulation of small molecule metabolic process | 0.009 | 167.150 | 0.006 |
| GO:1903578 | Biological Process | regulation of ATP metabolic process | 0.014 | 262.333 | 0.004 |
| GO:0006953 | Biological Process | acute-phase response | 0.021 | 401.872 | 0.002 |
| GO:0050714 | Biological Process | positive regulation of protein secretion | 0.007 | 128.490 | 0.008 |
| GO:1903531 | Biological Process | negative regulation of secretion by cell | 0.007 | 124.263 | 0.008 |
| GO:0046434 | Biological Process | organophosphate catabolic process | 0.004 | 80.718 | 0.012 |
| GO:0090087 | Biological Process | regulation of peptide transport | 0.005 | 93.505 | 0.011 |
| GO:0043255 | Biological Process | regulation of carbohydrate biosynthetic process | 0.010 | 187.010 | 0.005 |
| GO:0002791 | Biological Process | regulation of peptide secretion | 0.005 | 94.440 | 0.011 |
| GO:1903409 | Biological Process | reactive oxygen species biosynthetic process | 0.017 | 320.136 | 0.003 |
| GO:0090276 | Biological Process | regulation of peptide hormone secretion | 0.005 | 95.878 | 0.010 |
| GO:0051341 | Biological Process | regulation of oxidoreductase activity | 0.014 | 262.333 | 0.004 |
| GO:0045598 | Biological Process | regulation of fat cell differentiation | 0.007 | 129.370 | 0.008 |
| GO:0046887 | Biological Process | positive regulation of hormone secretion | 0.007 | 131.167 | 0.008 |
| GO:0010827 | Biological Process | regulation of glucose transmembrane transport | 0.013 | 242.154 | 0.004 |
| GO:0043470 | Biological Process | regulation of carbohydrate catabolic process | 0.016 | 309.639 | 0.003 |
| GO:0045913 | Biological Process | positive regulation of carbohydrate metabolic process | 0.013 | 245.299 | 0.004 |
| GO:0044042 | Biological Process | glucan metabolic process | 0.013 | 245.299 | 0.004 |
| GO:0002698 | Biological Process | negative regulation of immune effector process | 0.008 | 156.099 | 0.006 |
| GO:0005977 | Biological Process | glycogen metabolic process | 0.013 | 251.840 | 0.004 |
| GO:0019217 | Biological Process | regulation of fatty acid metabolic process | 0.010 | 190.788 | 0.005 |
| GO:0046777 | Biological Process | protein autophosphorylation | 0.005 | 98.375 | 0.010 |
| GO:0045833 | Biological Process | negative regulation of lipid metabolic process | 0.009 | 168.643 | 0.006 |
| GO:0045824 | Biological Process | negative regulation of innate immune response | 0.011 | 214.636 | 0.005 |
| GO:0050873 | Biological Process | brown fat cell differentiation | 0.019 | 349.778 | 0.003 |
| GO:0051092 | Biological Process | positive regulation of NF-kappaB transcription factor activity | 0.007 | 133.957 | 0.007 |
| GO:0006112 | Biological Process | energy reserve metabolic process | 0.011 | 212.225 | 0.005 |
| GO:1902176 | Biological Process | negative regulation of oxidative stress-induced intrinsic apoptotic signaling pathway | 0.029 | 555.529 | 0.002 |
| GO:0045730 | Biological Process | respiratory burst | 0.024 | 460.683 | 0.002 |
| GO:0006110 | Biological Process | regulation of glycolytic process | 0.020 | 370.353 | 0.003 |
| GO:0042311 | Biological Process | vasodilation | 0.021 | 393.500 | 0.003 |
| GO:0050994 | Biological Process | regulation of lipid catabolic process | 0.015 | 286.182 | 0.003 |
| GO:0051353 | Biological Process | positive regulation of oxidoreductase activity | 0.023 | 439.256 | 0.002 |
| GO:0000271 | Biological Process | polysaccharide biosynthetic process | 0.015 | 286.182 | 0.003 |
| GO:0002532 | Biological Process | production of molecular mediator involved in inflammatory response | 0.009 | 178.189 | 0.006 |
| GO:0046323 | Biological Process | glucose import | 0.013 | 242.154 | 0.004 |
| GO:1901292 | Biological Process | nucleoside phosphate catabolic process | 0.006 | 107.318 | 0.009 |
| GO:1903426 | Biological Process | regulation of reactive oxygen species biosynthetic process | 0.022 | 419.733 | 0.002 |
| GO:0051785 | Biological Process | positive regulation of nuclear division | 0.016 | 309.639 | 0.003 |
| GO:0006111 | Biological Process | regulation of gluconeogenesis | 0.019 | 363.231 | 0.003 |
| GO:0010907 | Biological Process | positive regulation of glucose metabolic process | 0.023 | 429.273 | 0.002 |
| GO:0032770 | Biological Process | positive regulation of monooxygenase activity | 0.036 | 674.571 | 0.001 |
| GO:0022898 | Biological Process | regulation of transmembrane transporter activity | 0.004 | 82.480 | 0.012 |
| GO:0106027 | Biological Process | neuron projection organization | 0.011 | 205.304 | 0.005 |
| GO:0009166 | Biological Process | nucleotide catabolic process | 0.006 | 113.102 | 0.009 |
| GO:0032768 | Biological Process | regulation of monooxygenase activity | 0.023 | 439.256 | 0.002 |
| GO:0050806 | Biological Process | positive regulation of synaptic transmission | 0.006 | 106.712 | 0.009 |
| GO:0045912 | Biological Process | negative regulation of carbohydrate metabolic process | 0.018 | 337.286 | 0.003 |
| GO:0045840 | Biological Process | positive regulation of mitotic nuclear division | 0.023 | 429.273 | 0.002 |
| GO:0046324 | Biological Process | regulation of glucose import | 0.017 | 314.800 | 0.003 |
| GO:0002793 | Biological Process | positive regulation of peptide secretion | 0.009 | 167.150 | 0.006 |
| GO:0097061 | Biological Process | dendritic spine organization | 0.012 | 233.185 | 0.004 |
| GO:0032409 | Biological Process | regulation of transporter activity | 0.004 | 72.646 | 0.014 |
| GO:1903580 | Biological Process | positive regulation of ATP metabolic process | 0.031 | 590.250 | 0.002 |
| GO:0060291 | Biological Process | long-term synaptic potentiation | 0.009 | 174.889 | 0.006 |
| GO:0090277 | Biological Process | positive regulation of peptide hormone secretion | 0.009 | 170.162 | 0.006 |
| GO:0045981 | Biological Process | positive regulation of nucleotide metabolic process | 0.026 | 497.053 | 0.002 |
| GO:1900544 | Biological Process | positive regulation of purine nucleotide metabolic process | 0.026 | 497.053 | 0.002 |
| GO:0002679 | Biological Process | respiratory burst involved in defense response | 0.059 | 1111.059 | 0.001 |
| GO:0010828 | Biological Process | positive regulation of glucose transmembrane transport | 0.023 | 429.273 | 0.002 |
| GO:0050709 | Biological Process | negative regulation of protein secretion | 0.014 | 255.243 | 0.004 |
| GO:0002673 | Biological Process | regulation of acute inflammatory response | 0.021 | 393.500 | 0.003 |
| GO:0009250 | Biological Process | glucan biosynthetic process | 0.022 | 410.609 | 0.002 |
| GO:0005978 | Biological Process | glycogen biosynthetic process | 0.022 | 410.609 | 0.002 |
| GO:2000378 | Biological Process | negative regulation of reactive oxygen species metabolic process | 0.021 | 393.500 | 0.003 |
| GO:1900078 | Biological Process | positive regulation of cellular response to insulin stimulus | 0.038 | 726.462 | 0.001 |
| GO:0050999 | Biological Process | regulation of nitric-oxide synthase activity | 0.036 | 674.571 | 0.001 |
| GO:0032881 | Biological Process | regulation of polysaccharide metabolic process | 0.022 | 419.733 | 0.002 |
| GO:0032885 | Biological Process | regulation of polysaccharide biosynthetic process | 0.026 | 497.053 | 0.002 |
| GO:0046628 | Biological Process | positive regulation of insulin receptor signaling pathway | 0.042 | 787.000 | 0.001 |
| GO:0045821 | Biological Process | positive regulation of glycolytic process | 0.050 | 944.400 | 0.001 |
| GO:0051000 | Biological Process | positive regulation of nitric-oxide synthase activity | 0.059 | 1111.059 | 0.001 |
| GO:0043954 | Biological Process | cellular component maintenance | 0.014 | 262.333 | 0.004 |
| GO:0046326 | Biological Process | positive regulation of glucose import | 0.027 | 510.486 | 0.002 |
| GO:0005980 | Biological Process | glycogen catabolic process | 0.067 | 1259.200 | 0.001 |
| GO:0031983 | Cellular Component | vesicle lumen | 0.003 | 61.025 | 0.016 |
| GO:0060205 | Cellular Component | cytoplasmic vesicle lumen | 0.003 | 61.212 | 0.016 |
| GO:0034774 | Cellular Component | secretory granule lumen | 0.003 | 61.783 | 0.016 |
| GO:0005788 | Cellular Component | endoplasmic reticulum lumen | 0.003 | 63.559 | 0.016 |
| GO:0030133 | Cellular Component | transport vesicle | 0.002 | 45.839 | 0.022 |
| GO:0005793 | Cellular Component | endoplasmic reticulum-Golgi intermediate compartment | 0.008 | 149.579 | 0.007 |
| GO:0005796 | Cellular Component | Golgi lumen | 0.009 | 187.679 | 0.005 |
| GO:0033116 | Cellular Component | endoplasmic reticulum-Golgi intermediate compartment membrane | 0.012 | 245.605 | 0.004 |
| GO:0002020 | Molecular Function | protease binding | 0.007 | 132.300 | 0.008 |
| GO:0005179 | Molecular Function | hormone activity | 0.008 | 147.000 | 0.007 |
| GO:0005158 | Molecular Function | insulin receptor binding | 0.045 | 841.909 | 0.001 |

| **Table S4. The intersection KEGG phenotype of the early CKM between the KEGG analysis based on the INS and the phenotypes related to the cobalt in the CTD** | | | | | | |
| --- | --- | --- | --- | --- | --- | --- |
| category | subcategory | ID | Description | Rich  Factor | Fold  Enrichment | pvalue |
| Organismal Systems | Excretory system | hsa04960 | Aldosterone-regulated sodium reabsorption | 0.026 | 224.816 | 0.004 |
| Human Diseases | Endocrine and metabolic disease | hsa04940 | Type I diabetes mellitus | 0.023 | 194.159 | 0.005 |
| Human Diseases | Endocrine and metabolic disease | hsa04930 | Type II diabetes mellitus | 0.021 | 181.766 | 0.006 |
| Organismal Systems | Endocrine system | hsa04913 | Ovarian steroidogenesis | 0.019 | 164.288 | 0.006 |
| Organismal Systems | Endocrine system | hsa04923 | Regulation of lipolysis in adipocytes | 0.017 | 144.797 | 0.007 |
| Organismal Systems | Aging | hsa04213 | Longevity regulating pathway - multiple species | 0.016 | 137.790 | 0.007 |
| Organismal Systems | Endocrine system | hsa04917 | Prolactin signaling pathway | 0.014 | 120.324 | 0.008 |
| Organismal Systems | Endocrine system | hsa04911 | Insulin secretion | 0.012 | 99.337 | 0.010 |
| Organismal Systems | Aging | hsa04211 | Longevity regulating pathway | 0.011 | 94.922 | 0.011 |
| Human Diseases | Cancer: specific types | hsa05215 | Prostate cancer | 0.010 | 87.173 | 0.011 |
| Human Diseases | Endocrine and metabolic disease | hsa04931 | Insulin resistance | 0.009 | 78.376 | 0.013 |
| Environmental Information Processing | Signal transduction | hsa04066 | HIF-1 signaling pathway | 0.009 | 77.664 | 0.013 |
| Organismal Systems | Endocrine system | hsa04914 | Progesterone-mediated oocyte maturation | 0.009 | 76.964 | 0.013 |
| Environmental Information Processing | Signal transduction | hsa04152 | AMPK signaling pathway | 0.008 | 70.025 | 0.014 |
| Environmental Information Processing | Signal transduction | hsa04068 | FoxO signaling pathway | 0.008 | 64.233 | 0.016 |
| Organismal Systems | Endocrine system | hsa04910 | Insulin signaling pathway | 0.007 | 61.906 | 0.016 |
| Cellular Processes | Cell growth and death | hsa04114 | Oocyte meiosis | 0.007 | 61.460 | 0.016 |
| Environmental Information Processing | Signal transduction | hsa04072 | Phospholipase D signaling pathway | 0.007 | 57.336 | 0.017 |
| Human Diseases | Endocrine and metabolic disease | hsa04932 | Non-alcoholic fatty liver disease | 0.006 | 54.414 | 0.018 |
| Environmental Information Processing | Signal transduction | hsa04150 | mTOR signaling pathway | 0.006 | 54.070 | 0.018 |
| Environmental Information Processing | Signal transduction | hsa04022 | cGMP-PKG signaling pathway | 0.006 | 51.464 | 0.019 |
| Cellular Processes | Transport and catabolism | hsa04140 | Autophagy - animal | 0.006 | 50.550 | 0.020 |
| Environmental Information Processing | Signal transduction | hsa04015 | Rap1 signaling pathway | 0.005 | 40.297 | 0.025 |
| Cellular Processes | Cell motility | hsa04810 | Regulation of actin cytoskeleton | 0.004 | 36.823 | 0.027 |
| Environmental Information Processing | Signal transduction | hsa04014 | Ras signaling pathway | 0.004 | 35.895 | 0.028 |
| Environmental Information Processing | Signal transduction | hsa04010 | MAPK signaling pathway | 0.003 | 28.477 | 0.035 |
| Environmental Information Processing | Signal transduction | hsa04151 | PI3K-Akt signaling pathway | 0.003 | 23.599 | 0.042 |
| Human Diseases | Neurodegenerative disease | hsa05010 | Alzheimer disease | 0.003 | 21.849 | 0.046 |

| **Table S5. The intersection GO phenotype of the late CKM between the GO analysis based on the identified gene (NOS3, AGT, ACE, CCL2, ICAM1, NPPB, SELE, PPARGC1A, GRK5) and the phenotypes related to the cobalt in the CTD** | | | | | | |
| --- | --- | --- | --- | --- | --- | --- |
| Common ID | Ontology | Highest GO Level | GO Term Name | Rich  Factor | Fold  Enrichment | pvalue |
| GO:0006979 | BP | 3 | response to oxidative stress | 0.005 | 10.546 | 0.014 |
| GO:2001233 | BP | 4 | regulation of apoptotic signaling pathway | 0.008 | 15.899 | 0.001 |
| GO:0031667 | BP | 4 | response to nutrient levels | 0.004 | 8.566 | 0.021 |
| GO:0062197 | BP | 4 | cellular response to chemical stress | 0.006 | 13.496 | 0.009 |
| GO:0045785 | BP | 3 | positive regulation of cell adhesion | 0.004 | 8.654 | 0.021 |
| GO:0043434 | BP | 4 | response to peptide hormone | 0.005 | 9.583 | 0.017 |
| GO:0016032 | BP | 1 | viral process | 0.005 | 9.807 | 0.017 |
| GO:0019693 | BP | 4 | ribose phosphate metabolic process | 0.004 | 8.744 | 0.021 |
| GO:0034599 | BP | 4 | cellular response to oxidative stress | 0.008 | 16.925 | 0.006 |
| GO:1901653 | BP | 5 | cellular response to peptide | 0.008 | 16.745 | 0.001 |
| GO:0009259 | BP | 5 | ribonucleotide metabolic process | 0.004 | 9.007 | 0.019 |
| GO:0009150 | BP | 6 | purine ribonucleotide metabolic process | 0.004 | 9.411 | 0.018 |
| GO:1901342 | BP | 4 | regulation of vasculature development | 0.006 | 11.595 | 0.012 |
| GO:2001234 | BP | 4 | negative regulation of apoptotic signaling pathway | 0.008 | 17.202 | 0.006 |
| GO:0045765 | BP | 4 | regulation of angiogenesis | 0.006 | 11.890 | 0.011 |
| GO:0071375 | BP | 5 | cellular response to peptide hormone stimulus | 0.006 | 13.496 | 0.009 |
| GO:0097191 | BP | 4 | extrinsic apoptotic signaling pathway | 0.013 | 27.374 | 0.000 |
| GO:0050878 | BP | 3 | regulation of body fluid levels | 0.005 | 11.344 | 0.013 |
| GO:0032102 | BP | 3 | negative regulation of response to external stimulus | 0.004 | 9.205 | 0.019 |
| GO:0072593 | BP | 3 | reactive oxygen species metabolic process | 0.008 | 17.636 | 0.005 |
| GO:0001558 | BP | 3 | regulation of cell growth | 0.005 | 10.041 | 0.016 |
| GO:0090130 | BP | 2 | tissue migration | 0.005 | 10.959 | 0.013 |
| GO:0010631 | BP | 4 | epithelial cell migration | 0.005 | 11.193 | 0.013 |
| GO:0043410 | BP | 5 | positive regulation of MAPK cascade | 0.004 | 8.836 | 0.020 |
| GO:0062012 | BP | 3 | regulation of small molecule metabolic process | 0.006 | 12.681 | 0.010 |
| GO:0090132 | BP | 3 | epithelium migration | 0.005 | 11.104 | 0.013 |
| GO:0051402 | BP | 4 | neuron apoptotic process | 0.007 | 14.374 | 0.008 |
| GO:0050900 | BP | 2 | leukocyte migration | 0.008 | 15.899 | 0.001 |
| GO:0007159 | BP | 4 | leukocyte cell-cell adhesion | 0.007 | 15.026 | 0.001 |
| GO:0019058 | BP | 2 | viral life cycle | 0.006 | 13.158 | 0.009 |
| GO:0002237 | BP | 4 | response to molecule of bacterial origin | 0.011 | 22.750 | 0.000 |
| GO:0003018 | BP | 4 | vascular process in circulatory system | 0.015 | 30.526 | 0.000 |
| GO:0032496 | BP | 4 | response to lipopolysaccharide | 0.011 | 24.123 | 0.000 |
| GO:0043523 | BP | 5 | regulation of neuron apoptotic process | 0.008 | 17.202 | 0.006 |
| GO:0006869 | BP | 4 | lipid transport | 0.004 | 9.205 | 0.019 |
| GO:1903037 | BP | 5 | regulation of leukocyte cell-cell adhesion | 0.005 | 10.988 | 0.013 |
| GO:0022409 | BP | 4 | positive regulation of cell-cell adhesion | 0.006 | 12.915 | 0.010 |
| GO:2001236 | BP | 5 | regulation of extrinsic apoptotic signaling pathway | 0.019 | 40.102 | 0.000 |
| GO:0050727 | BP | 4 | regulation of inflammatory response | 0.005 | 9.876 | 0.016 |
| GO:0070371 | BP | 5 | ERK1 and ERK2 cascade | 0.006 | 12.643 | 0.010 |
| GO:0060537 | BP | 4 | muscle tissue development | 0.005 | 9.583 | 0.017 |
| GO:0043542 | BP | 5 | endothelial cell migration | 0.007 | 14.676 | 0.008 |
| GO:0048511 | BP | 1 | rhythmic process | 0.007 | 14.132 | 0.008 |
| GO:0072001 | BP | 4 | renal system development | 0.006 | 12.758 | 0.010 |
| GO:0051051 | BP | 2 | negative regulation of transport | 0.004 | 9.085 | 0.019 |
| GO:0034329 | BP | 5 | cell junction assembly | 0.004 | 9.266 | 0.018 |
| GO:1901293 | BP | 5 | nucleoside phosphate biosynthetic process | 0.010 | 21.342 | 0.000 |
| GO:0071216 | BP | 3 | cellular response to biotic stimulus | 0.008 | 15.839 | 0.007 |
| GO:0001822 | BP | 4 | kidney development | 0.006 | 13.158 | 0.009 |
| GO:0009165 | BP | 6 | nucleotide biosynthetic process | 0.010 | 21.488 | 0.000 |
| GO:0034612 | BP | 5 | response to tumor necrosis factor | 0.008 | 15.959 | 0.007 |
| GO:1903039 | BP | 5 | positive regulation of leukocyte cell-cell adhesion | 0.007 | 15.153 | 0.007 |
| GO:0010632 | BP | 3 | regulation of epithelial cell migration | 0.007 | 14.085 | 0.008 |
| GO:0033002 | BP | 3 | muscle cell proliferation | 0.008 | 16.722 | 0.006 |
| GO:0034762 | BP | 3 | regulation of transmembrane transport | 0.004 | 8.584 | 0.021 |
| GO:0070372 | BP | 6 | regulation of ERK1 and ERK2 cascade | 0.006 | 13.628 | 0.009 |
| GO:0003012 | BP | 3 | muscle system process | 0.004 | 9.145 | 0.019 |
| GO:0061458 | BP | 4 | reproductive system development | 0.006 | 13.496 | 0.009 |
| GO:0002685 | BP | 3 | regulation of leukocyte migration | 0.013 | 27.374 | 0.000 |
| GO:0048608 | BP | 3 | reproductive structure development | 0.007 | 13.672 | 0.009 |
| GO:0072522 | BP | 5 | purine-containing compound biosynthetic process | 0.011 | 23.848 | 0.000 |
| GO:0002443 | BP | 3 | leukocyte mediated immunity | 0.004 | 8.950 | 0.020 |
| GO:0046390 | BP | 5 | ribose phosphate biosynthetic process | 0.009 | 18.014 | 0.005 |
| GO:0006164 | BP | 6 | purine nucleotide biosynthetic process | 0.012 | 24.594 | 0.000 |
| GO:0048659 | BP | 4 | smooth muscle cell proliferation | 0.011 | 23.581 | 0.003 |
| GO:0048660 | BP | 4 | regulation of smooth muscle cell proliferation | 0.011 | 24.123 | 0.003 |
| GO:1904019 | BP | 5 | epithelial cell apoptotic process | 0.014 | 28.553 | 0.002 |
| GO:0071219 | BP | 4 | cellular response to molecule of bacterial origin | 0.008 | 17.636 | 0.005 |
| GO:0009743 | BP | 4 | response to carbohydrate | 0.008 | 17.562 | 0.005 |
| GO:0010634 | BP | 4 | positive regulation of epithelial cell migration | 0.011 | 23.714 | 0.003 |
| GO:0071222 | BP | 5 | cellular response to lipopolysaccharide | 0.009 | 18.655 | 0.005 |
| GO:0009260 | BP | 6 | ribonucleotide biosynthetic process | 0.009 | 18.572 | 0.005 |
| GO:0045216 | BP | 5 | cell-cell junction organization | 0.009 | 19.706 | 0.004 |
| GO:0045444 | BP | 4 | fat cell differentiation | 0.008 | 16.925 | 0.006 |
| GO:0043269 | BP | 4 | regulation of monoatomic ion transport | 0.006 | 13.396 | 0.001 |
| GO:0010959 | BP | 5 | regulation of metal ion transport | 0.008 | 15.980 | 0.001 |
| GO:0042593 | BP | 4 | glucose homeostasis | 0.012 | 24.403 | 0.000 |
| GO:0048771 | BP | 2 | tissue remodeling | 0.016 | 33.489 | 0.000 |
| GO:0033500 | BP | 3 | carbohydrate homeostasis | 0.012 | 24.309 | 0.000 |
| GO:0030324 | BP | 4 | lung development | 0.010 | 21.748 | 0.004 |
| GO:0060541 | BP | 4 | respiratory system development | 0.009 | 19.079 | 0.005 |
| GO:0007548 | BP | 3 | sex differentiation | 0.007 | 14.574 | 0.008 |
| GO:0009152 | BP | 7 | purine ribonucleotide biosynthetic process | 0.009 | 19.893 | 0.004 |
| GO:0030323 | BP | 4 | respiratory tube development | 0.010 | 21.306 | 0.004 |
| GO:2001237 | BP | 5 | negative regulation of extrinsic apoptotic signaling pathway | 0.020 | 41.973 | 0.001 |
| GO:0008406 | BP | 4 | gonad development | 0.009 | 18.014 | 0.005 |
| GO:0002687 | BP | 3 | positive regulation of leukocyte migration | 0.013 | 28.170 | 0.002 |
| GO:0045137 | BP | 3 | development of primary sexual characteristics | 0.008 | 17.636 | 0.005 |
| GO:0006816 | BP | 7 | calcium ion transport | 0.007 | 14.710 | 0.001 |
| GO:0043524 | BP | 5 | negative regulation of neuron apoptotic process | 0.012 | 25.909 | 0.003 |
| GO:0014823 | BP | 2 | response to activity | 0.027 | 56.721 | 0.001 |
| GO:0060485 | BP | 4 | mesenchyme development | 0.006 | 12.492 | 0.010 |
| GO:0003015 | BP | 4 | heart process | 0.012 | 24.215 | 0.000 |
| GO:0010594 | BP | 4 | regulation of endothelial cell migration | 0.009 | 17.937 | 0.005 |
| GO:0097746 | BP | 5 | blood vessel diameter maintenance | 0.026 | 55.228 | 0.000 |
| GO:0035296 | BP | 5 | regulation of tube diameter | 0.026 | 55.228 | 0.000 |
| GO:0034284 | BP | 5 | response to monosaccharide | 0.009 | 19.522 | 0.004 |
| GO:0035150 | BP | 4 | regulation of tube size | 0.026 | 54.867 | 0.000 |
| GO:0070374 | BP | 6 | positive regulation of ERK1 and ERK2 cascade | 0.009 | 19.522 | 0.004 |
| GO:0051090 | BP | 3 | regulation of DNA-binding transcription factor activity | 0.005 | 11.017 | 0.013 |
| GO:0010595 | BP | 5 | positive regulation of endothelial cell migration | 0.015 | 32.041 | 0.002 |
| GO:0008625 | BP | 5 | extrinsic apoptotic signaling pathway via death domain receptors | 0.024 | 49.968 | 0.001 |
| GO:0051924 | BP | 6 | regulation of calcium ion transport | 0.012 | 25.490 | 0.000 |
| GO:0070555 | BP | 5 | response to interleukin-1 | 0.014 | 30.415 | 0.002 |
| GO:1903522 | BP | 4 | regulation of blood circulation | 0.011 | 24.031 | 0.000 |
| GO:0060047 | BP | 5 | heart contraction | 0.012 | 25.285 | 0.000 |
| GO:0062013 | BP | 3 | positive regulation of small molecule metabolic process | 0.014 | 29.559 | 0.002 |
| GO:0071674 | BP | 3 | mononuclear cell migration | 0.010 | 20.375 | 0.004 |
| GO:0008217 | BP | 3 | regulation of blood pressure | 0.021 | 44.891 | 0.000 |
| GO:0090257 | BP | 4 | regulation of muscle system process | 0.008 | 17.132 | 0.006 |
| GO:0007043 | BP | 6 | cell-cell junction assembly | 0.013 | 26.906 | 0.002 |
| GO:0030100 | BP | 4 | regulation of endocytosis | 0.007 | 14.424 | 0.008 |
| GO:0009746 | BP | 6 | response to hexose | 0.010 | 20.179 | 0.004 |
| GO:1904035 | BP | 6 | regulation of epithelial cell apoptotic process | 0.017 | 36.499 | 0.001 |
| GO:1901888 | BP | 4 | regulation of cell junction assembly | 0.009 | 19.522 | 0.004 |
| GO:0009749 | BP | 7 | response to glucose | 0.010 | 20.677 | 0.004 |
| GO:0045807 | BP | 3 | positive regulation of endocytosis | 0.013 | 27.080 | 0.002 |
| GO:0043500 | BP | 2 | muscle adaptation | 0.017 | 35.272 | 0.001 |
| GO:0008016 | BP | 5 | regulation of heart contraction | 0.014 | 30.269 | 0.000 |
| GO:0072577 | BP | 6 | endothelial cell apoptotic process | 0.029 | 61.725 | 0.000 |
| GO:0051345 | BP | 5 | positive regulation of hydrolase activity | 0.004 | 8.708 | 0.021 |
| GO:0051091 | BP | 4 | positive regulation of DNA-binding transcription factor activity | 0.009 | 18.249 | 0.005 |
| GO:0050886 | BP | 3 | endocrine process | 0.032 | 67.699 | 0.000 |
| GO:0071322 | BP | 5 | cellular response to carbohydrate stimulus | 0.012 | 26.070 | 0.002 |
| GO:1900542 | BP | 5 | regulation of purine nucleotide metabolic process | 0.022 | 46.125 | 0.001 |
| GO:0006140 | BP | 4 | regulation of nucleotide metabolic process | 0.022 | 45.623 | 0.001 |
| GO:0001678 | BP | 4 | cellular glucose homeostasis | 0.018 | 37.928 | 0.000 |
| GO:0097696 | BP | 4 | receptor signaling pathway via STAT | 0.010 | 21.976 | 0.003 |
| GO:2000351 | BP | 7 | regulation of endothelial cell apoptotic process | 0.032 | 67.699 | 0.000 |
| GO:1902041 | BP | 6 | regulation of extrinsic apoptotic signaling pathway via death domain receptors | 0.042 | 87.444 | 0.000 |
| GO:0071326 | BP | 6 | cellular response to monosaccharide stimulus | 0.013 | 27.982 | 0.002 |
| GO:0045123 | BP | 3 | cellular extravasation | 0.040 | 83.947 | 0.000 |
| GO:0003073 | BP | 4 | regulation of systemic arterial blood pressure | 0.042 | 87.444 | 0.000 |
| GO:0071331 | BP | 7 | cellular response to hexose stimulus | 0.014 | 28.360 | 0.002 |
| GO:0007259 | BP | 5 | receptor signaling pathway via JAK-STAT | 0.011 | 24.123 | 0.003 |
| GO:0071333 | BP | 5 | cellular response to glucose stimulus | 0.014 | 28.947 | 0.002 |
| GO:0034405 | BP | 3 | response to fluid shear stress | 0.059 | 123.451 | 0.000 |
| GO:0071548 | BP | 5 | response to dexamethasone | 0.023 | 47.697 | 0.021 |
| GO:0043502 | BP | 3 | regulation of muscle adaptation | 0.021 | 44.652 | 0.001 |
| GO:1990776 | BP | 5 | response to angiotensin | 0.045 | 95.394 | 0.000 |
| GO:0043271 | BP | 3 | negative regulation of monoatomic ion transport | 0.015 | 31.559 | 0.002 |
| GO:1904646 | BP | 6 | cellular response to amyloid-beta | 0.023 | 48.806 | 0.020 |
| GO:0048662 | BP | 4 | negative regulation of smooth muscle cell proliferation | 0.028 | 59.117 | 0.000 |
| GO:0042311 | BP | 6 | vasodilation | 0.042 | 87.444 | 0.000 |
| GO:0072676 | BP | 4 | lymphocyte migration | 0.016 | 33.579 | 0.002 |
| GO:0051353 | BP | 5 | positive regulation of oxidoreductase activity | 0.023 | 48.806 | 0.020 |
| GO:0003014 | BP | 3 | renal system process | 0.016 | 33.050 | 0.002 |
| GO:0010742 | BP | 5 | macrophage derived foam cell differentiation | 0.025 | 52.467 | 0.019 |
| GO:0090077 | BP | 4 | foam cell differentiation | 0.024 | 51.187 | 0.019 |
| GO:0060218 | BP | 5 | hematopoietic stem cell differentiation | 0.028 | 58.296 | 0.017 |
| GO:0010743 | BP | 4 | regulation of macrophage derived foam cell differentiation | 0.030 | 63.596 | 0.016 |
| GO:0001974 | BP | 3 | blood vessel remodeling | 0.059 | 123.451 | 0.000 |
| GO:1902042 | BP | 6 | negative regulation of extrinsic apoptotic signaling pathway via death domain receptors | 0.065 | 135.398 | 0.000 |
| GO:0019882 | BP | 2 | antigen processing and presentation | 0.017 | 35.875 | 0.001 |
| GO:0006525 | BP | 6 | arginine metabolic process | 0.053 | 110.456 | 0.009 |
| GO:0010907 | BP | 4 | positive regulation of glucose metabolic process | 0.023 | 47.697 | 0.021 |
| GO:0042310 | BP | 6 | vasoconstriction | 0.022 | 47.161 | 0.001 |
| GO:2000352 | BP | 7 | negative regulation of endothelial cell apoptotic process | 0.028 | 58.296 | 0.017 |
| GO:0071604 | BP | 3 | transforming growth factor beta production | 0.023 | 47.697 | 0.021 |
| GO:1904037 | BP | 6 | positive regulation of epithelial cell apoptotic process | 0.023 | 47.697 | 0.021 |
| GO:1901889 | BP | 4 | negative regulation of cell junction assembly | 0.031 | 65.583 | 0.015 |
| GO:1904385 | BP | 6 | cellular response to angiotensin | 0.056 | 116.593 | 0.000 |
| GO:0035929 | BP | 4 | steroid hormone secretion | 0.037 | 77.728 | 0.013 |
| GO:0034616 | BP | 4 | response to laminar fluid shear stress | 0.071 | 149.905 | 0.007 |
| GO:0090183 | BP | 3 | regulation of kidney development | 0.029 | 61.725 | 0.016 |
| GO:0051385 | BP | 6 | response to mineralocorticoid | 0.025 | 52.467 | 0.019 |
| GO:0071634 | BP | 4 | regulation of transforming growth factor beta production | 0.024 | 51.187 | 0.019 |
| GO:0003176 | BP | 5 | aortic valve development | 0.023 | 48.806 | 0.020 |
| GO:1903580 | BP | 6 | positive regulation of ATP metabolic process | 0.031 | 65.583 | 0.015 |
| GO:0000305 | BP | 4 | response to oxygen radical | 0.036 | 74.952 | 0.013 |
| GO:0003203 | BP | 5 | endocardial cushion morphogenesis | 0.024 | 51.187 | 0.019 |
| GO:0045981 | BP | 4 | positive regulation of nucleotide metabolic process | 0.053 | 110.456 | 0.000 |
| GO:1900544 | BP | 5 | positive regulation of purine nucleotide metabolic process | 0.053 | 110.456 | 0.000 |
| GO:0044060 | BP | 4 | regulation of endocrine process | 0.025 | 52.467 | 0.019 |
| GO:0019229 | BP | 5 | regulation of vasoconstriction | 0.029 | 60.831 | 0.000 |
| GO:0030728 | BP | 3 | ovulation | 0.063 | 131.167 | 0.008 |
| GO:0019430 | BP | 4 | removal of superoxide radicals | 0.045 | 95.394 | 0.010 |
| GO:0010744 | BP | 4 | positive regulation of macrophage derived foam cell differentiation | 0.053 | 110.456 | 0.009 |
| GO:0061337 | BP | 6 | cardiac conduction | 0.020 | 42.397 | 0.001 |
| GO:0006527 | BP | 5 | arginine catabolic process | 0.100 | 209.867 | 0.005 |
| GO:0010544 | BP | 4 | negative regulation of platelet activation | 0.056 | 116.593 | 0.009 |
| GO:0051926 | BP | 4 | negative regulation of calcium ion transport | 0.029 | 61.725 | 0.000 |
| GO:0000303 | BP | 5 | response to superoxide | 0.037 | 77.728 | 0.013 |
| GO:0003044 | BP | 5 | regulation of systemic arterial blood pressure mediated by a chemical signal | 0.064 | 133.957 | 0.000 |
| GO:0071450 | BP | 5 | cellular response to oxygen radical | 0.042 | 87.444 | 0.011 |
| GO:0071451 | BP | 6 | cellular response to superoxide | 0.042 | 87.444 | 0.011 |
| GO:0045454 | BP | 3 | cell redox homeostasis | 0.024 | 51.187 | 0.019 |
| GO:0098742 | BP | 4 | cell-cell adhesion via plasma-membrane adhesion molecules | 0.007 | 15.044 | 0.007 |
| GO:0110096 | BP | 4 | cellular response to aldehyde | 0.059 | 123.451 | 0.008 |
| GO:2000831 | BP | 4 | regulation of steroid hormone secretion | 0.045 | 95.394 | 0.010 |
| GO:1900027 | BP | 6 | regulation of ruffle assembly | 0.032 | 67.699 | 0.015 |
| GO:0097178 | BP | 7 | ruffle assembly | 0.023 | 47.697 | 0.021 |
| GO:0035930 | BP | 5 | corticosteroid hormone secretion | 0.053 | 110.456 | 0.009 |
| GO:2000846 | BP | 5 | regulation of corticosteroid hormone secretion | 0.063 | 131.167 | 0.008 |
| GO:0002691 | BP | 4 | regulation of cellular extravasation | 0.048 | 99.937 | 0.000 |
| GO:0001990 | BP | 4 | regulation of systemic arterial blood pressure by hormone | 0.077 | 161.436 | 0.000 |
| GO:0072678 | BP | 5 | T cell migration | 0.027 | 56.721 | 0.001 |
| GO:0048873 | BP | 4 | homeostasis of number of cells within a tissue | 0.029 | 59.962 | 0.017 |
| GO:0014850 | BP | 3 | response to muscle activity | 0.071 | 149.905 | 0.000 |
| GO:2000353 | BP | 7 | positive regulation of endothelial cell apoptotic process | 0.040 | 83.947 | 0.012 |
| GO:0048246 | BP | 4 | macrophage chemotaxis | 0.025 | 52.467 | 0.019 |
| GO:1901186 | BP | 4 | positive regulation of ERBB signaling pathway | 0.032 | 67.699 | 0.015 |
| GO:1904044 | BP | 5 | response to aldosterone | 0.053 | 110.456 | 0.009 |
| GO:0032309 | BP | 5 | icosanoid secretion | 0.022 | 46.637 | 0.021 |
| GO:0003180 | BP | 4 | aortic valve morphogenesis | 0.026 | 55.228 | 0.018 |
| GO:0046320 | BP | 6 | regulation of fatty acid oxidation | 0.029 | 61.725 | 0.016 |
| GO:0034349 | BP | 5 | glial cell apoptotic process | 0.063 | 131.167 | 0.008 |
| GO:0070168 | BP | 3 | negative regulation of biomineral tissue development | 0.033 | 69.956 | 0.014 |
| GO:0098857 | CC | 3 | membrane microdomain | 0.010 | 23.106 | 0.000 |
| GO:0045121 | CC | 4 | membrane raft | 0.010 | 23.186 | 0.000 |
| GO:0009897 | CC | 3 | external side of plasma membrane | 0.007 | 16.374 | 0.001 |
| GO:0044853 | CC | 4 | plasma membrane raft | 0.018 | 39.123 | 0.001 |
| GO:0005901 | CC | 5 | caveola | 0.024 | 53.913 | 0.001 |
| GO:0001664 | MF | 4 | G protein-coupled receptor binding | 0.010 | 21.216 | 0.000 |
| GO:0005516 | MF | 3 | calmodulin binding | 0.010 | 20.078 | 0.004 |
| GO:0031406 | MF | 4 | carboxylic acid binding | 0.010 | 21.000 | 0.004 |
| GO:0043177 | MF | 3 | organic acid binding | 0.010 | 19.788 | 0.004 |
| GO:0005179 | MF | 5 | hormone activity | 0.016 | 32.667 | 0.002 |

| **Table S6. The intersection KEGG phenotype of the late CKM between the KEGG analysis identified gene (NOS3, AGT, ACE, CCL2, ICAM1, NPPB, SELE, PPARGC1A, GRK5) and the phenotypes related to the cobalt in the CTD** | | | | | | | |
| --- | --- | --- | --- | --- | --- | --- | --- |
| category | subcategory | ID | Description | Rich  Factor | Fold  Enrichment | pvalue | geneID |
| Human Diseases | Endocrine and metabolic disease | hsa04933 | AGE-RAGE signaling pathway in diabetic complications | 0.050 | 46.991 | 0.000 | 4846/183/6347/3383/6401 |
| Human Diseases | Cardiovascular disease | hsa05418 | Fluid shear stress and atherosclerosis | 0.028 | 26.928 | 0.000 | 4846/6347/3383/6401 |
| Human Diseases | Infectious disease: parasitic | hsa05144 | Malaria | 0.060 | 56.953 | 0.000 | 6347/3383/6401 |
| Human Diseases | Endocrine and metabolic disease | hsa04931 | Insulin resistance | 0.028 | 26.125 | 0.000 | 4846/183/10891 |
| Environmental Information Processing | Signal transduction | hsa04668 | TNF signaling pathway | 0.025 | 23.930 | 0.000 | 6347/3383/6401 |
| Organismal Systems | Endocrine system | hsa04614 | Renin-angiotensin system | 0.087 | 82.541 | 0.000 | 183/1636 |
| Human Diseases | Infectious disease: parasitic | hsa05143 | African trypanosomiasis | 0.054 | 51.309 | 0.001 | 3383/6401 |
| Organismal Systems | Endocrine system | hsa04924 | Renin secretion | 0.029 | 27.514 | 0.002 | 183/1636 |
| Human Diseases | Immune disease | hsa05323 | Rheumatoid arthritis | 0.021 | 19.984 | 0.004 | 6347/3383 |
| Human Diseases | Cardiovascular disease | hsa05410 | Hypertrophic cardiomyopathy | 0.020 | 19.176 | 0.005 | 183/1636 |
| Human Diseases | Infectious disease: parasitic | hsa05142 | Chagas disease | 0.019 | 18.431 | 0.005 | 1636/6347 |
| Organismal Systems | Circulatory system | hsa04270 | Vascular smooth muscle contraction | 0.015 | 14.167 | 0.008 | 183/4879 |
| Environmental Information Processing | Signal transduction | hsa04371 | Apelin signaling pathway | 0.014 | 13.560 | 0.009 | 4846/10891 |
| Environmental Information Processing | Signaling molecules and interaction | hsa04514 | Cell adhesion molecules | 0.013 | 11.865 | 0.012 | 3383/6401 |
| Environmental Information Processing | Signal transduction | hsa04022 | cGMP-PKG signaling pathway | 0.012 | 11.436 | 0.012 | 4846/4879 |
| Human Diseases | Infectious disease: viral | hsa05164 | Influenza A | 0.012 | 10.974 | 0.013 | 6347/3383 |

**References**

Aggarwal, R, J W Ostrominski and M Vaduganathan. 2024. Prevalence of Cardiovascular-Kidney-Metabolic Syndrome Stages in US Adults, 2011-2020. *Jama*. 331 (21): 1858-1860.

Ahmadi, M N, M Hamer, J M R Gill, M Murphy, J P Sanders, A Doherty and E Stamatakis. 2023. Brief bouts of device-measured intermittent lifestyle physical activity and its association with major adverse cardiovascular events and mortality in people who do not exercise: a prospective cohort study. *Lancet Public Health*. 8 (10): e800-e810.

Group, K D I G O K C W. 2024. KDIGO 2024 Clinical Practice Guideline for the Evaluation and Management of Chronic Kidney Disease. *Kidney Int*. 105 (4s): S117-s314.

Huang, Q, J Wan, W Nan, S Li, B He and Z Peng. 2024. Association between manganese exposure in heavy metals mixtures and the prevalence of sarcopenia in US adults from NHANES 2011-2018. *J Hazard Mater*. 464: 133005.

Khan, S S, K Matsushita, Y Sang, S H Ballew, M E Grams, A Surapaneni, M J Blaha, A P Carson, A R Chang, E Ciemins et al. 2024. Development and Validation of the American Heart Association's PREVENT Equations. *Circulation*. 149 (6): 430-449.
